# Correction: Year-Round Reproduction and Induced Spawning of Chinese Amphioxus, *Branchiostoma belcheri*, in Laboratory

**DOI:** 10.1371/journal.pone.0099264

**Published:** 2014-05-27

**Authors:** The PLOS ONE Staff

## Notice of Republication

This article was republished on May 9th, 2014, due to Figure 1, Figure 2, and Figure 3 being ordered incorrectly. Please download the PDF again to view the corrected article. The originally published, uncorrected article and the republished, corrected article are provided here for reference.

## Supporting Information

File S1 Originally published, uncorrected article.
(PDF)

File S2 Republished, corrected article.
(PDF)
