# Supplementary figures and images for: Year-Round Reproduction and Induced Spawning of Chinese Amphioxus, Branchiostoma belcheri, in Laboratory
Source: PLoS One. 2013 Sep 26;8(9):e75461. doi: 10.1371/journal.pone.0075461 (PMC3784433; doi:10.1371/journal.pone.0075461)

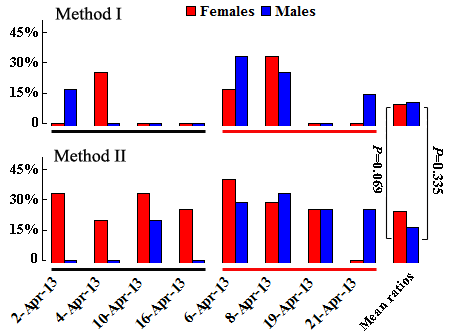

Supplement: Figure S1 — Side-by-side induction experiments using two different temperature-shift methods. Spawning percentage is the number of spawned animals (males or females separately) over the total number of temperature shocked animals (males or females separately) in each examined date. Inductions using Method I (shifting from 19°C to 27°C) and II (shifting from 22°C to 27°C) are shown separately. Inductions of the two cohorts of animals are respectively marked by black and red bold lines under the horizontal coordinates. Statistical analyses of the data are carried out using SPASS software (version 16.0) and two-tailed chi-squared test. (TIF) [file pone.0075461.s001.tif]
