# Supplementary material for: Year-Round Reproduction and Induced Spawning of Chinese Amphioxus, Branchiostoma belcheri, in Laboratory
Source: PLoS One. 2013 Sep 26;8(9):e75461. doi: 10.1371/journal.pone.0075461 (PMC3784433; doi:10.1371/journal.pone.0075461)
Supplement: Table S1 — Recurring spawning in B . japonicum reared under three different temperatures. The experiment was set on 24th Nov. 2011 and data collection was started from 6th Feb. 2012 when most animals began to develop their gonads. On each data collecting date, animal number in each reproductive phase was recorded. Abbreviations: Dev, Development; Spa, Spawning. (DOC) [file pone.0075461.s002.doc]

**Table S1. Recurring spawning in *B. japonicum* reared under three different temperatures.**

| **Date** | **18-19˚C** | | | | | | **Room temperature** | | | | | | **25-27˚C** | | | | | |
| --- | --- | --- | --- | --- | --- | --- | --- | --- | --- | --- | --- | --- | --- | --- | --- | --- | --- | --- |
|  | **Undeveloped** | **1^st^ Dev** | **1^st^ Spa** | **2^nd^ Dev** | **2^nd^ Spa** | **Total No.** | **Undeveloped** | **1^st^ Dev** | **1^st^ Spa** | **2^nd^ Dev** | **2^nd^ Spa** | **Total No.** | **Undeveloped** | **1^st^ Dev** | **1^st^ Spa** | **2^nd^ Dev** | **2^nd^ Spa** | **Total No.** |
| 6-Feb-12 | 20 | 58 |  |  |  | 78 | 11 | 77 |  |  |  | 88 | 5 | 82 |  |  |  | 87 |
| 26-Feb-12 | 8 | 70 |  |  |  | 78 | 1 | 87 |  |  |  | 88 | 1 | 45 | 41 |  |  | 87 |
| 20-Mar-12 | 0 | 67 | 11 |  |  | 78 | 0 | 75 | 13 |  |  | 88 | 1 | 24 | 19 | 32 | 11 | 87 |
| 7-Apr-12 |  | 31 | 47 |  |  | 78 |  | 10 | 75 |  |  | 85 | 1 | 3 | 39 | 6 | 38 | 87 |
| 19-Apr-12 |  | 9 | 69 |  |  | 78 |  |  | 85 |  |  | 85 | 1 | 3 | 39 | 6 | 38 | 87 |
| 4-May-12 |  | 8 | 69 |  |  | 77 |  |  | 69 | 16 |  | 85 |  |  | 42 |  | 42 | 84 |
| 18-May-12 |  | 8 | 69 |  |  | 77 |  |  | 69 | 1 | 15 | 85 |  |  | 39 |  | 42 | 81 |
| 6-Jun-12 |  | 8 | 69 |  |  | 77 |  |  | 69 |  | 16 | 85 |  |  | 39 |  | 42 | 81 |
| 26-Jun-12 |  | 8 | 69 |  |  | 77 |  |  | 69 |  | 16 | 85 |  |  | 39 |  | 42 | 81 |
| 26-Jul-12 |  | 8 | 69 |  |  | 77 |  |  | 69 |  | 16 | 85 |  |  | 39 |  | 42 | 81 |
| **Since all animals of the three groups have no gonadal development within the last two months, we mixed and redistrubed each group of animals, and re-started our records after 26^th^, Jul. 2013.** | | | | | | | | | | | | | | | | | | |
| 30-Aug-12 | 58 | 19 |  |  |  | 77 | 64 | 16 |  |  |  | 80 | 58 | 22 |  |  |  | 80 |
| 14-Sep-12 | 58 | 19 |  |  |  | 77 | 63 | 16 |  |  |  | 79 | 57 | 23 |  |  |  | 80 |
| 29-Sep-12 | 45 | 32 |  |  |  | 77 | 55 | 24 |  |  |  | 79 | 36 | 44 |  |  |  | 80 |
| 14-Oct-12 | 43 | 34 |  |  |  | 77 | 47 | 32 |  |  |  | 79 | 29 | 51 |  |  |  | 80 |
| 29-Oct-12 | 24 | 53 |  |  |  | 77 | 30 | 49 |  |  |  | 79 | 9 | 70 |  |  |  | 79 |
| 13-Nov-12 | 12 | 65 |  |  |  | 77 | 26 | 53 |  |  |  | 79 | 7 | 70 |  |  |  | 77 |
| 28-Nov-12 | 12 | 65 |  |  |  | 77 | 17 | 62 |  |  |  | 79 | 6 | 70 |  |  |  | 76 |
| 20-Dec-12 | 11 | 66 |  |  |  | 77 | 13 | 66 |  |  |  | 79 | 5 | 71 |  |  |  | 76 |
| 3-Jan-13 | 11 | 66 |  |  |  | 77 | 13 | 66 |  |  |  | 79 | 5 | 71 |  |  |  | 76 |
| 19-Jan-13 | 4 | 71 | 2 |  |  | 77 | 8 | 71 |  |  |  | 79 | 5 | 71 |  |  |  | 76 |
| 2-Feb-13 | 4 | 68 | 5 |  |  | 77 | 8 | 71 |  |  |  | 79 | 5 | 66 | 4 |  |  | 75 |
| 21-Feb-13 | 2 | 66 | 9 |  |  | 77 | 4 | 75 |  |  |  | 79 | 3 | 46 | 26 |  |  | 75 |
| 7-Mar-13 | 2 | 66 | 9 |  |  | 77 | 2 | 77 |  |  |  | 79 | 3 | 36 | 22 | 13 |  | 74 |
| 22-Mar-13 | 2 | 66 | 9 |  |  | 77 | 2 | 70 | 7 |  |  | 79 | 2 | 13 | 41 | 17 |  | 73 |

Note: The experiment was set on 24^th^ Nov. 2011 and data collection was started from 6^th^ Feb. 2012 when most animals began to develop their gonads. On each data collecting date, animal number in each **reproductive phase was recorded.** Abbreviations: Dev, Development; Spa, Spawning.
