# Supplementary material for: Year-Round Reproduction and Induced Spawning of Chinese Amphioxus, Branchiostoma belcheri, in Laboratory
Source: PLoS One. 2013 Sep 26;8(9):e75461. doi: 10.1371/journal.pone.0075461 (PMC3784433; doi:10.1371/journal.pone.0075461)
Supplement: Table S2 — Side-by-side spawning induction using two methods on two cohorts of B . becheri animals. (DOC) [file pone.0075461.s003.doc]

**Table S2. Side-by-side spawning induction using two methods on two cohorts of *B.becheri* animals.**

| Animal cohorts | Induction Date | 19°C to 27°C | | | | | | | | 22°C to 27°C | | | | | | | |
| --- | --- | --- | --- | --- | --- | --- | --- | --- | --- | --- | --- | --- | --- | --- | --- | --- | --- |
|  |  | Number of spontaneously spawned animals | | Number of induced animals | | Number of induced spawning animals | | Induced spawning ratio | | Number of spontaneously spawned animals | | Number of induced animals | | Number of induced spawning animals | | Induced spawning ratio | |
|  |  | Female | Male | Female | Male | Female | Male | Female | Male | Female | Male | Female | Male | Female | Male | Female | Male |
| The first cohort of animals collected on March, 26 | 2-Apr-13 | 0 | 0 | 4 | 6 | 0 | 1 | 0.0% | 16.7% | 7 | 11 | 6 | 4 | 2 | 0 | 33.3% | 0.0% |
|  | 4-Apr-13 | 0 | 0 | 4 | 2 | 1 | 0 | 25.0% | 0.0% | 0 | 0 | 5 | 1 | 1 | 0 | 20.0% | 0.0% |
|  | 10-Apr-13 | 0 | 1 | 4 | 6 | 0 | 0 | 0.0% | 0.0% | 0 | 1 | 3 | 5 | 1 | 1 | 33.3% | 20.0% |
|  | 16-Apr-13 | 0 | 0 | 4 | 6 | 0 | 0 | 0.0% | 0.0% | 0 | 0 | 4 | 6 | 1 | 0 | 25.0% | 0.0% |
| The second cohort of animals collected on April, 1 | 6-Apr-13 | 0 | 0 | 6 | 6 | 1 | 2 | 16.7% | 33.3% | 1 | 0 | 5 | 7 | 2 | 2 | 40.0% | 28.6% |
|  | 8-Apr-13 | 0 | 0 | 6 | 4 | 2 | 1 | 33.3% | 25.0% | 0 | 0 | 7 | 3 | 2 | 1 | 28.6% | 33.3% |
|  | 19-Apr-13 | 0 | 1 | 6 | 6 | 0 | 0 | 0.0% | 0.0% | 2 | 4 | 4 | 4 | 1 | 1 | 25.0% | 25.0% |
|  | 21-Apr-13 | 0 | 0 | 4 | 7 | 0 | 1 | 0.0% | 14.3% | 0 | 0 | 4 | 4 | 0 | 1 | 0.0% | 25.0% |
