# Supplementary material for: Year-Round Reproduction and Induced Spawning of Chinese Amphioxus, Branchiostoma belcheri, in Laboratory
Source: PLoS One. 2013 Sep 26;8(9):e75461. doi: 10.1371/journal.pone.0075461 (PMC3784433; doi:10.1371/journal.pone.0075461)
Supplement: Table S3 — Spawning induction of B . belcheri using Method I and II. Period from 7th June 2011 to 31st October 2011 includes spawning induction records of animals which are reared under Zhang’s conditions and shocked using Method I; period from 1st November 2012 to 11th April 2012 includes spawning induction records of animals which are reared under updated conditions and shocked using Method I; and period from 12th April 2012 to 23nd Oct. 2012 includes spawning induction records of animals reared under updated conditions and shocked using Method II. (DOC) [file pone.0075461.s004.doc]

**Table S3. Spawning induction of *B. belcheri* using Method I and II.**

| **Date** | **Number of induced animals** | | **Number of spawned animals** | | **Effeciency** | |
| --- | --- | --- | --- | --- | --- | --- |
|  | **Female** | **Male** | **Female** | **Male** | **Female** | **Male** |
| **2011-6-7** | **13** | **10** | **1** | **0** | **7.7%** | **0.0%** |
| **2011-6-9** | **10** | **10** | **0** | **0** | **0.0%** | **0.0%** |
| **2011-6-10** | **10** | **8** | **0** | **0** | **0.0%** | **0.0%** |
| **2011-6-11** | **10** | **10** | **0** | **0** | **0.0%** | **0.0%** |
| **2011-6-12** | **10** | **10** | **0** | **0** | **0.0%** | **0.0%** |
| **2011-6-27** | **10** | **10** | **0** | **1** | **0.0%** | **10.0%** |
| **2011-6-29** | **10** | **10** | **0** | **0** | **0.0%** | **0.0%** |
| **2011-6-30** | **10** | **10** | **0** | **0** | **0.0%** | **0.0%** |
| **2011-7-2** | **15** | **18** | **0** | **0** | **0.0%** | **0.0%** |
| **2011-7-20** | **12** | **13** | **0** | **1** | **0.0%** | **7.7%** |
| **2011-7-25** | **12** | **13** | **1** | **2** | **8.3%** | **15.4%** |
| **2011-7-28** | **17** | **18** | **3** | **3** | **17.6%** | **16.7%** |
| **2011-7-29** | **14** | **15** | **0** | **0** | **0.0%** | **0.0%** |
| **2011-7-30** | **14** | **15** | **1** | **2** | **7.1%** | **13.3%** |
| **2011-8-1** | **13** | **13** | **0** | **0** | **0.0%** | **0.0%** |
| **2011-8-5** | **20** | **20** | **0** | **0** | **0.0%** | **0.0%** |
| **2011-8-9** | **20** | **20** | **0** | **0** | **0.0%** | **0.0%** |
| **2011-8-10** | **20** | **20** | **0** | **0** | **0.0%** | **0.0%** |
| **2011-8-11** | **22** | **23** | **0** | **0** | **0.0%** | **0.0%** |
| **2011-8-12** | **22** | **23** | **0** | **0** | **0.0%** | **0.0%** |
| **2011-8-13** | **20** | **20** | **0** | **0** | **0.0%** | **0.0%** |
| **2011-8-14** | **20** | **20** | **0** | **0** | **0.0%** | **0.0%** |
| **2011-8-15** | **20** | **20** | **0** | **0** | **0.0%** | **0.0%** |
| **2011-8-17** | **15** | **15** | **0** | **0** | **0.0%** | **0.0%** |
| **2011-8-18** | **15** | **15** | **0** | **0** | **0.0%** | **0.0%** |
| **2011-8-19** | **15** | **15** | **0** | **0** | **0.0%** | **0.0%** |
| **2011-8-20** | **13** | **14** | **1** | **0** | **7.7%** | **0.0%** |
| **2011-8-22** | **13** | **14** | **0** | **0** | **0.0%** | **0.0%** |
| **2011-8-23** | **15** | **15** | **0** | **1** | **0.0%** | **6.7%** |
| **2011-8-24** | **20** | **20** | **0** | **0** | **0.0%** | **0.0%** |
| **2011-8-25** | **15** | **15** | **1** | **0** | **6.7%** | **0.0%** |
| **2011-8-26** | **15** | **15** | **0** | **1** | **0.0%** | **6.7%** |
| **2011-8-27** | **20** | **20** | **0** | **0** | **0.0%** | **0.0%** |
| **2011-8-28** | **20** | **20** | **5** | **10** | **25.0%** | **50.0%** |
| **2011-8-29** | **20** | **20** | **1** | **2** | **5.0%** | **10.0%** |
| **2011-8-30** | **19** | **18** | **0** | **0** | **0.0%** | **0.0%** |
| **2011-8-31** | **15** | **15** | **0** | **0** | **0.0%** | **0.0%** |
| **2011-9-1** | **25** | **25** | **1** | **1** | **4.0%** | **4.0%** |
| **2011-9-2** | **15** | **15** | **1** | **1** | **6.7%** | **6.7%** |
| **2011-9-3** | **22** | **23** | **0** | **0** | **0.0%** | **0.0%** |
| **2011-9-4** | **14** | **15** | **0** | **0** | **0.0%** | **0.0%** |
| **2011-9-5** | **22** | **23** | **1** | **2** | **4.5%** | **8.7%** |
| **2011-9-6** | **12** | **13** | **4** | **6** | **33.3%** | **46.2%** |
| **2011-9-7** | **12** | **13** | **8** | **3** | **66.7%** | **23.1%** |
| **2011-9-8** | **17** | **18** | **0** | **1** | **0.0%** | **5.6%** |
| **2011-9-9** | **17** | **18** | **10** | **6** | **58.8%** | **33.3%** |
| **2011-9-10** | **5** | **6** | **1** | **0** | **20.0%** | **0.0%** |
| **2011-9-11** | **17** | **18** | **1** | **1** | **5.9%** | **5.6%** |
| **2011-9-13** | **15** | **15** | **0** | **0** | **0.0%** | **0.0%** |
| **2011-9-14** | **15** | **15** | **0** | **3** | **0.0%** | **20.0%** |
| **2011-9-15** | **17** | **18** | **3** | **2** | **17.6%** | **11.1%** |
| **2011-9-16** | **10** | **13** | **0** | **0** | **0.0%** | **0.0%** |
| **2011-9-17** | **19** | **20** | **2** | **1** | **10.5%** | **5.0%** |
| **2011-9-20** | **9** | **10** | **0** | **0** | **0.0%** | **0.0%** |
| **2011-9-21** | **17** | **18** | **1** | **1** | **5.9%** | **5.6%** |
| **2011-9-22** | **14** | **17** | **0** | **0** | **0.0%** | **0.0%** |
| **2011-9-23** | **17** | **18** | **1** | **0** | **5.9%** | **0.0%** |
| **2011-9-24** | **17** | **18** | **1** | **1** | **5.9%** | **5.6%** |
| **2011-9-25** | **8** | **10** | **0** | **0** | **0.0%** | **0.0%** |
| **2011-9-26** | **14** | **14** | **0** | **0** | **0.0%** | **0.0%** |
| **2011-9-27** | **17** | **18** | **0** | **0** | **0.0%** | **0.0%** |
| **2011-9-28** | **15** | **15** | **0** | **0** | **0.0%** | **0.0%** |
| **2011-9-29** | **13** | **13** | **0** | **0** | **0.0%** | **0.0%** |
| **2011-10-1** | **10** | **10** | **6** | **3** | **60.0%** | **30.0%** |
| **2011-10-2** | **29** | **30** | **0** | **0** | **0.0%** | **0.0%** |
| **2011-10-3** | **10** | **10** | **0** | **0** | **0.0%** | **0.0%** |
| **2011-10-4** | **13** | **13** | **0** | **0** | **0.0%** | **0.0%** |
| **2011-10-5** | **19** | **19** | **0** | **0** | **0.0%** | **0.0%** |
| **2011-10-9** | **10** | **10** | **0** | **0** | **0.0%** | **0.0%** |
| **2011-10-10** | **12** | **13** | **0** | **1** | **0.0%** | **7.7%** |
| **2011-10-11** | **10** | **11** | **0** | **0** | **0.0%** | **0.0%** |
| **2011-10-14** | **10** | **11** | **0** | **0** | **0.0%** | **0.0%** |
| **2011-10-17** | **10** | **13** | **0** | **0** | **0.0%** | **0.0%** |
| **2011-10-18** | **20** | **21** | **1** | **0** | **5.0%** | **0.0%** |
| **2011-10-19** | **20** | **20** | **10** | **5** | **50.0%** | **25.0%** |
| **2011-10-20** | **20** | **23** | **0** | **0** | **0.0%** | **0.0%** |
| **2011-10-21** | **15** | **22** | **1** | **1** | **6.7%** | **4.5%** |
| **2011-10-22** | **18** | **18** | **1** | **0** | **5.6%** | **0.0%** |
| **2011-10-24** | **7** | **8** | **0** | **0** | **0.0%** | **0.0%** |
| **2011-10-25** | **7** | **8** | **1** | **0** | **14.3%** | **0.0%** |
| **2011-10-26** | **15** | **16** | **1** | **1** | **6.7%** | **6.3%** |
| **2011-10-27** | **11** | **11** | **0** | **0** | **0.0%** | **0.0%** |
| **2011-10-28** | **14** | **15** | **0** | **0** | **0.0%** | **0.0%** |
| **2011-10-31** | **11** | **11** | **1** | **0** | **9.1%** | **0.0%** |
| 2011-11-1 | 10 | 11 | 0 | 0 | 0.0% | 0.0% |
| 2011-11-2 | 11 | 10 | 5 | 3 | 45.5% | 30.0% |
| 2011-11-3 | 8 | 8 | 0 | 0 | 0.0% | 0.0% |
| 2011-11-4 | 10 | 10 | 0 | 0 | 0.0% | 0.0% |
| 2011-11-7 | 9 | 9 | 0 | 0 | 0.0% | 0.0% |
| 2011-11-8 | 10 | 10 | 1 | 1 | 10.0% | 10.0% |
| 2011-11-9 | 10 | 10 | 0 | 0 | 0.0% | 0.0% |
| 2011-11-10 | 8 | 9 | 0 | 0 | 0.0% | 0.0% |
| 2011-11-15 | 8 | 8 | 1 | 4 | 12.5% | 50.0% |
| 2011-11-16 | 5 | 6 | 0 | 0 | 0.0% | 0.0% |
| 2011-11-17 | 11 | 11 | 3 | 4 | 27.3% | 36.4% |
| 2011-11-18 | 14 | 14 | 5 | 4 | 35.7% | 28.6% |
| 2011-11-21 | 11 | 11 | 0 | 0 | 0.0% | 0.0% |
| 2011-11-22 | 11 | 11 | 2 | 1 | 18.2% | 9.1% |
| 2011-11-23 | 10 | 10 | 3 | 3 | 30.0% | 30.0% |
| 2011-11-24 | 9 | 9 | 4 | 4 | 44.4% | 44.4% |
| 2011-11-28 | 8 | 8 | 0 | 1 | 0.0% | 12.5% |
| 2011-11-29 | 13 | 13 | 0 | 1 | 0.0% | 7.7% |
| 2011-11-30 | 16 | 16 | 2 | 2 | 12.5% | 12.5% |
| 2011-12-1 | 12 | 13 | 1 | 1 | 8.3% | 7.7% |
| 2011-12-3 | 25 | 25 | 5 | 6 | 20.0% | 24.0% |
| 2011-12-6 | 11 | 11 | 0 | 4 | 0.0% | 36.4% |
| 2011-12-7 | 10 | 11 | 1 | 0 | 10.0% | 0.0% |
| 2011-12-8 | 10 | 10 | 3 | 0 | 30.0% | 0.0% |
| 2011-12-10 | 8 | 8 | 0 | 1 | 0.0% | 12.5% |
| 2011-12-13 | 10 | 10 | 0 | 1 | 0.0% | 10.0% |
| 2011-12-15 | 9 | 9 | 3 | 3 | 33.3% | 33.3% |
| 2011-12-17 | 8 | 9 | 0 | 0 | 0.0% | 0.0% |
| 2011-12-20 | 7 | 8 | 2 | 0 | 28.6% | 0.0% |
| 2011-12-21 | 13 | 14 | 0 | 1 | 0.0% | 7.1% |
| 2011-12-22 | 13 | 13 | 0 | 0 | 0.0% | 0.0% |
| 2011-12-24 | 13 | 14 | 2 | 1 | 15.4% | 7.1% |
| 2011-12-27 | 8 | 8 | 1 | 0 | 12.5% | 0.0% |
| 2011-12-31 | 12 | 12 | 2 | 1 | 16.7% | 8.3% |
| 2012-1-4 | 17 | 7 | 3 | 1 | 17.6% | 14.3% |
| 2012-1-7 | 3 | 33 | 0 | 2 | 0.0% | 6.1% |
| 2012-1-9 | 25 | 25 | 0 | 0 | 0.0% | 0.0% |
| 2012-1-10 | 20 | 20 | 0 | 0 | 0.0% | 0.0% |
| 2012-2-9 | 10 | 5 | 0 | 0 | 0.0% | 0.0% |
| 2012-2-15 | 13 | 12 | 1 | 2 | 7.7% | 16.7% |
| 2012-2-22 | 14 | 14 | 6 | 1 | 42.9% | 7.1% |
| 2012-2-23 | 14 | 15 | 2 | 2 | 14.3% | 13.3% |
| 2012-2-25 | 15 | 15 | 0 | 1 | 0.0% | 6.7% |
| 2012-2-27 | 20 | 20 | 3 | 3 | 15.0% | 15.0% |
| 2012-2-28 | 11 | 11 | 0 | 0 | 0.0% | 0.0% |
| 2012-2-29 | 11 | 12 | 0 | 0 | 0.0% | 0.0% |
| 2012-3-1 | 12 | 13 | 0 | 0 | 0.0% | 0.0% |
| 2012-3-2 | 11 | 11 | 0 | 0 | 0.0% | 0.0% |
| 2012-3-3 | 15 | 15 | 0 | 0 | 0.0% | 0.0% |
| 2012-3-6 | 15 | 15 | 1 | 1 | 6.7% | 6.7% |
| 2012-3-7 | 11 | 12 | 0 | 0 | 0.0% | 0.0% |
| 2012-3-8 | 15 | 15 | 0 | 0 | 0.0% | 0.0% |
| 2012-3-9 | 15 | 15 | 1 | 0 | 6.7% | 0.0% |
| 2012-3-12 | 12 | 13 | 3 | 0 | 25.0% | 0.0% |
| 2012-3-13 | 15 | 15 | 0 | 2 | 0.0% | 13.3% |
| 2012-3-14 | 13 | 14 | 1 | 0 | 7.7% | 0.0% |
| 2012-3-15 | 12 | 12 | 0 | 0 | 0.0% | 0.0% |
| 2012-3-17 | 8 | 11 | 4 | 4 | 50.0% | 36.4% |
| 2012-3-20 | 8 | 10 | 0 | 0 | 0.0% | 0.0% |
| 2012-3-22 | 14 | 16 | 3 | 0 | 21.4% | 0.0% |
| 2012-3-23 | 10 | 12 | 0 | 0 | 0.0% | 0.0% |
| 2012-3-27 | 14 | 16 | 0 | 0 | 0.0% | 0.0% |
| 2012-3-28 | 14 | 16 | 4 | 5 | 28.6% | 31.3% |
| 2012-4-5 | 15 | 22 | 2 | 2 | 13.3% | 9.1% |
| 2012-4-6 | 12 | 13 | 0 | 0 | 0.0% | 0.0% |
| 2012-4-7 | 9 | 10 | 0 | 0 | 0.0% | 0.0% |
| 2012-4-9 | 10 | 12 | 0 | 0 | 0.0% | 0.0% |
| 2012-4-10 | 17 | 20 | 0 | 0 | 0.0% | 0.0% |
| 2012-4-11 | 20 | 30 | 0 | 0 | 0.0% | 0.0% |
| **2012-4-12** | **20** | **25** | **4** | **1** | **20.0%** | **4.0%** |
| **2012-4-13** | **10** | **12** | **0** | **0** | **0.0%** | **0.0%** |
| **2012-4-14** | **20** | **20** | **1** | **0** | **5.0%** | **0.0%** |
| **2012-4-16** | **18** | **17** | **7** | **7** | **38.9%** | **41.2%** |
| **2012-4-17** | **10** | **10** | **6** | **8** | **60.0%** | **80.0%** |
| **2012-4-18** | **18** | **7** | **13** | **4** | **72.2%** | **57.1%** |
| **2012-4-19** | **11** | **7** | **6** | **4** | **54.5%** | **57.1%** |
| **2012-4-20** | **4** | **3** | **1** | **3** | **25.0%** | **100.0%** |
| **2012-4-21** | **13** | **8** | **0** | **0** | **0.0%** | **0.0%** |
| **2012-4-23** | **6** | **3** | **3** | **1** | **50.0%** | **33.3%** |
| **2012-4-24** | **8** | **4** | **2** | **0** | **25.0%** | **0.0%** |
| **2012-4-25** | **6** | **3** | **2** | **2** | **33.3%** | **66.7%** |
| **2012-4-26** | **15** | **6** | **1** | **2** | **6.7%** | **33.3%** |
| **2012-4-27** | **8** | **2** | **4** | **1** | **50.0%** | **50.0%** |
| **2012-4-28** | **6** | **4** | **0** | **0** | **0.0%** | **0.0%** |
| **2012-5-2** | **6** | **2** | **3** | **0** | **50.0%** | **0.0%** |
| **2012-5-5** | **6** | **2** | **4** | **2** | **66.7%** | **100.0%** |
| **2012-5-6** | **9** | **5** | **5** | **1** | **55.6%** | **20.0%** |
| **2012-5-7** | **2** | **1** | **1** | **0** | **50.0%** | **0.0%** |
| **2012-5-8** | **4** | **3** | **1** | **0** | **25.0%** | **0.0%** |
| **2012-5-9** | **7** | **7** | **2** | **1** | **28.6%** | **14.3%** |
| **2012-5-10** | **13** | **13** | **0** | **0** | **0.0%** | **0.0%** |
| **2012-5-16** | **5** | **3** | **3** | **1** | **60.0%** | **33.3%** |
| **2012-5-18** | **3** | **3** | **0** | **2** | **0.0%** | **66.7%** |
| **2012-5-21** | **7** | **4** | **1** | **2** | **14.3%** | **50.0%** |
| **2012-5-22** | **12** | **6** | **6** | **5** | **50.0%** | **83.3%** |
| **2012-5-24** | **5** | **5** | **2** | **0** | **40.0%** | **0.0%** |
| **2012-5-26** | **6** | **8** | **1** |  | **16.7%** | **0.0%** |
| **2012-5-28** | **5** | **5** | **0** | **0** | **0.0%** | **0.0%** |
| **2012-5-30** | **4** | **2** | **0** | **0** | **0.0%** | **0.0%** |
| **2012-5-31** | **8** | **3** | **3** | **0** | **37.5%** | **0.0%** |
| **2012-6-1** | **4** | **5** | **0** | **0** | **0.0%** | **0.0%** |
| **2012-6-2** | **14** | **13** | **0** | **0** | **0.0%** | **0.0%** |
| **2012-6-8** | **5** | **3** | **0** | **0** | **0.0%** | **0.0%** |
| **2012-6-12** | **19** | **11** | **5** | **1** | **26.3%** | **9.1%** |
| **2012-6-14** | **5** | **5** | **0** | **2** | **0.0%** | **40.0%** |
| **2012-6-16** | **12** | **8** | **7** | **1** | **58.3%** | **12.5%** |
| **2012-6-18** | **10** | **5** | **0** | **0** | **0.0%** | **0.0%** |
| **2012-6-20** | **9** | **11** | **0** | **0** | **0.0%** | **0.0%** |
| **2012-6-22** | **13** | **8** | **1** | **1** | **7.7%** | **12.5%** |
| **2012-6-24** | **5** | **4** | **0** | **0** | **0.0%** | **0.0%** |
| **2012-6-27** | **9** | **6** | **2** | **0** | **22.2%** | **0.0%** |
| **2012-6-29** | **14** | **15** | **4** | **3** | **28.6%** | **20.0%** |
| **2012-7-1** | **10** | **4** | **8** | **2** | **80.0%** | **50.0%** |
| **2012-7-3** | **9** | **8** | **5** | **0** | **55.6%** | **0.0%** |
| **2012-7-4** | **7** | **7** | **2** | **1** | **28.6%** | **14.3%** |
| **2012-7-8** | **19** | **15** | **7** | **4** | **36.8%** | **26.7%** |
| **2012-7-10** | **9** | **4** | **2** | **2** | **22.2%** | **50.0%** |
| **2012-7-11** | **9** | **7** | **5** | **3** | **55.6%** | **42.9%** |
| **2012-7-13** | **6** | **4** | **2** | **2** | **33.3%** | **50.0%** |
| **2012-7-15** | **6** | **4** | **2** | **2** | **33.3%** | **50.0%** |
| **2012-7-17** | **7** | **5** | **4** | **1** | **57.1%** | **20.0%** |
| **2012-7-18** | **9** | **3** | **3** | **0** | **33.3%** | **0.0%** |
| **2012-7-20** | **10** | **9** | **3** | **4** | **30.0%** | **44.4%** |
| **2012-7-22** | **8** | **6** | **3** | **1** | **37.5%** | **16.7%** |
| **2012-7-24** | **5** | **4** | **1** | **1** | **20.0%** | **25.0%** |
| **2012-7-25** | **4** | **5** | **1** | **0** | **25.0%** | **0.0%** |
| **2012-7-27** | **7** | **7** | **3** | **2** | **42.9%** | **28.6%** |
| **2012-7-29** | **6** | **8** | **1** | **1** | **16.7%** | **12.5%** |
| **2012-7-31** | **7** | **7** | **0** | **2** | **0.0%** | **28.6%** |
| **2012-8-1** | **3** | **6** | **0** | **0** | **0.0%** | **0.0%** |
| **2012-8-3** | **10** | **6** | **2** | **0** | **20.0%** | **0.0%** |
| **2012-8-5** | **12** | **6** | **1** | **1** | **8.3%** | **16.7%** |
| **2012-8-7** | **8** | **5** | **3** | **0** | **37.5%** | **0.0%** |
| **2012-8-8** | **9** | **4** | **2** | **1** | **22.2%** | **25.0%** |
| **2012-8-10** | **8** | **6** | **2** | **3** | **25.0%** | **50.0%** |
| **2012-8-12** | **7** | **4** | **4** | **2** | **57.1%** | **50.0%** |
| **2012-8-14** | **6** | **3** | **2** | **3** | **33.3%** | **100.0%** |
| **2012-8-19** | **10** | **8** | **3** | **4** | **30.0%** | **50.0%** |
| **2012-8-20** | **7** | **6** | **0** | **1** | **0.0%** | **16.7%** |
| **2012-8-22** | **10** | **11** | **2** | **3** | **20.0%** | **27.3%** |
| **2012-8-23** | **8** | **8** | **0** | **2** | **0.0%** | **25.0%** |
| **2012-8-26** | **3** | **4** | **1** | **4** | **33.3%** | **100.0%** |
| **2012-8-28** | **4** | **4** | **2** | **2** | **50.0%** | **50.0%** |
| **2012-8-29** | **4** | **3** | **3** | **0** | **75.0%** | **0.0%** |
| **2012-8-31** | **5** | **7** | **0** | **0** | **0.0%** | **0.0%** |
| **2012-9-1** | **7** | **5** | **0** | **0** | **0.0%** | **0.0%** |
| **2012-9-2** | **8** | **8** | **0** | **1** | **0.0%** | **12.5%** |
| **2012-9-3** | **7** | **9** | **0** | **0** | **0.0%** | **0.0%** |
| **2012-9-4** | **11** | **8** | **0** | **1** | **0.0%** | **12.5%** |
| **2012-9-5** | **10** | **8** | **1** | **1** | **10.0%** | **12.5%** |
| **2012-9-6** | **9** | **9** | **2** | **1** | **22.2%** | **11.1%** |
| **2012-9-7** | **5** | **3** | **1** | **1** | **20.0%** | **33.3%** |
| **2012-9-9** | **4** | **4** | **0** | **0** | **0.0%** | **0.0%** |
| **2012-9-10** | **5** | **5** | **0** | **2** | **0.0%** | **40.0%** |
| **2012-9-11** | **13** | **5** | **1** | **0** | **7.7%** | **0.0%** |
| **2012-9-26** | **7** | **9** | **4** | **3** | **57.1%** | **33.3%** |
| **2012-9-27** | **6** | **4** | **2** | **1** | **33.3%** | **25.0%** |
| **2012-9-29** | **6** | **5** | **1** | **0** | **16.7%** | **0.0%** |
| **2012-10-9** | **6** | **6** | **1** | **0** | **16.7%** | **0.0%** |
| **2012-10-10** | **5** | **3** | **5** | **2** | **100.0%** | **66.7%** |
| **2012-10-12** | **5** | **3** | **3** | **0** | **60.0%** | **0.0%** |
| **2012-10-13** | **5** | **3** | **1** | **1** | **20.0%** | **33.3%** |
| **2012-10-15** | **5** | **5** | **1** | **0** | **20.0%** | **0.0%** |
| **2012-10-17** | **5** | **6** | **1** | **2** | **20.0%** | **33.3%** |
| **2012-10-19** | **5** | **2** | **0** | **0** | **0.0%** | **0.0%** |
| **2012-10-20** | **6** | **4** | **1** | **0** | **16.7%** | **0.0%** |
| **2012-10-22** | **5** | **4** | **1** | **1** | **20.0%** | **25.0%** |
| **2012-10-23** | **7** | **5** | **1** | **1** | **14.3%** | **20.0%** |

Note: Period from 7^th^ June 2011 to 31^st^ Oct. 2011 includes spawning induction records of animals which are reared under Zhang’s conditions and shocked using Method I; period from 1^st^ Nov. 2012 to 11^th^ April 2012 includes spawning induction records of animals which are reared under updated conditions and shocked using Method I; and period from 12^th^ April 2012 to 23^nd^ Oct. 2012 includes spawning induction records of animals reared under updated conditions and shocked using Method II.
