# Supplementary material for: Year-Round Reproduction and Induced Spawning of Chinese Amphioxus, Branchiostoma belcheri, in Laboratory
Source: PLoS One. 2013 Sep 26;8(9):e75461. doi: 10.1371/journal.pone.0075461 (PMC3784433; doi:10.1371/journal.pone.0075461)
Supplement: Table S4 — Spawning induction records of B . japonicum in 2011 and 2012. (DOC) [file pone.0075461.s005.doc]

**Table S4. Spawning induction records of *B. japonicum* in 2011 and 2012.**

| **Date** | **Number of induced animals** | | **Number of spawned animals** | | **Effeciency** | |
| --- | --- | --- | --- | --- | --- | --- |
|  | **Female** | **Male** | **Female** | **Male** | **Female** | **Male** |
| 10-Mar-11 | 21 | 26 | 1 | 5 | 4.8% | 19.2% |
| 12-Mar-11 | 15 | 15 | 0 | 1 | 0.0% | 6.7% |
| 16-Mar-11 | 25 | 25 | 4 | 10 | 16.0% | 40.0% |
| 17-Mar-11 | 25 | 25 | 7 | 14 | 28.0% | 56.0% |
| 18-Mar-11 | 25 | 20 | 0 | 4 | 0.0% | 20.0% |
| 19-Mar-11 | 25 | 16 | 2 | 2 | 8.0% | 12.5% |
| 20-Mar-11 | 25 | 16 | 8 | 3 | 32.0% | 18.8% |
| 22-Mar-11 | 26 | 18 | 4 | 3 | 15.4% | 16.7% |
| 23-Mar-11 | 14 | 6 | 5 | 5 | 35.7% | 83.3% |
| 24-Mar-11 | 14 | 6 | 0 | 1 | 0.0% | 16.7% |
| 25-Mar-11 | 14 | 6 | 2 | 3 | 14.3% | 50.0% |
| 26-Mar-11 | 14 | 6 | 2 | 1 | 14.3% | 16.7% |
| 27-Mar-11 | 15 | 6 | 0 | 2 | 0.0% | 33.3% |
| 28-Mar-11 | 25 | 16 | 10 | 2 | 40.0% | 12.5% |
| 29-Mar-11 | 14 | 6 | 5 | 3 | 35.7% | 50.0% |
| 31-Mar-11 | 23 | 16 | 3 | 7 | 13.0% | 43.8% |
| 1-Apr-11 | 12 | 6 | 1 | 2 | 8.3% | 33.3% |
| 2-Apr-11 | 27 | 16 | 0 | 4 | 0.0% | 25.0% |
| 4-Apr-11 | 25 | 16 | 0 | 7 | 0.0% | 43.8% |
| 6-Apr-11 | 15 | 15 | 2 | 3 | 13.3% | 20.0% |
| 7-Apr-11 | 12 | 6 | 1 | 2 | 8.3% | 33.3% |
| 8-Apr-11 | 45 | 15 | 2 | 2 | 4.4% | 13.3% |
| 9-Apr-11 | 22 | 16 | 1 | 1 | 4.5% | 6.3% |
| 10-Apr-11 | 25 | 16 | 1 | 10 | 4.0% | 62.5% |
| 11-Apr-11 | 24 | 16 | 6 | 8 | 25.0% | 50.0% |
| 13-Apr-11 | 12 | 6 | 0 | 1 | 0.0% | 16.7% |
| 14-Apr-11 | 25 | 16 | 0 | 1 | 0.0% | 6.3% |
| 15-Apr-11 | 25 | 16 | 0 | 1 | 0.0% | 6.3% |
| 17-Apr-11 | 26 | 17 | 5 | 9 | 19.2% | 52.9% |
| 18-Apr-11 | 12 | 6 | 4 | 2 | 33.3% | 33.3% |
| 19-Apr-11 | 12 | 6 | 7 | 5 | 58.3% | 83.3% |
| 21-Apr-11 | 13 | 4 | 3 | 3 | 23.1% | 75.0% |
| 22-Apr-11 | 12 | 6 | 3 | 6 | 25.0% | 100.0% |
| 23-Apr-11 | 12 | 5 | 3 | 1 | 25.0% | 20.0% |
| 25-Apr-11 | 12 | 6 | 1 | 4 | 8.3% | 66.7% |
| 3-May-11 | 12 | 6 | 3 | 2 | 25.0% | 33.3% |
| 10-May-11 | 22 | 10 | 3 | 2 | 13.6% | 20.0% |
| 6-Apr-12 | 10 | 11 | 0 | 0 | 0.0% | 0.0% |
| 7-Apr-12 | 8 | 10 | 1 | 2 | 12.5% | 20.0% |
| 11-Apr-12 | 10 | 10 | 1 | 0 | 10.0% | 0.0% |
| 12-Apr-12 | 14 | 6 | 0 | 6 | 0.0% | 100.0% |
| 13-Apr-12 | 11 | 10 | 1 | 1 | 9.1% | 10.0% |
| 14-Apr-12 | 10 | 10 | 9 | 9 | 90.0% | 90.0% |
| 16-Apr-12 | 9 | 3 | 5 | 3 | 55.6% | 100.0% |
| 17-Apr-12 | 10 | 2 | 0 | 0 | 0.0% | 0.0% |
| 18-Apr-12 | 8 | 2 | 6 | 2 | 75.0% | 100.0% |
| 19-Apr-12 | 10 | 4 | 5 | 3 | 50.0% | 75.0% |
| 20-Apr-12 | 7 | 2 | 4 | 1 | 57.1% | 50.0% |
| 21-Apr-12 | 4 | 3 | 2 | 2 | 50.0% | 66.7% |
| 23-Apr-12 | 5 | 3 | 1 | 3 | 20.0% | 100.0% |
| 24-Apr-12 | 2 | 5 | 0 | 5 | 0.0% | 100.0% |
| 25-Apr-12 | 8 | 1 | 3 | 1 | 37.5% | 100.0% |
| 26-Apr-12 | 4 | 6 | 1 | 5 | 25.0% | 83.3% |
| 27-Apr-12 | 4 | 3 | 1 | 3 | 25.0% | 100.0% |
| 28-Apr-12 | 2 | 4 | 0 | 4 | 0.0% | 100.0% |
| 30-Apr-12 | 4 | 4 | 1 | 0 | 25.0% | 0.0% |
| 1-May-12 | 4 | 3 | 4 | 2 | 100.0% | 66.7% |
| 2-May-12 | 4 | 3 | 1 | 2 | 25.0% | 66.7% |
| 3-May-12 | 4 | 1 | 0 | 1 | 0.0% | 100.0% |
| 4-May-12 | 4 | 2 | 3 | 1 | 75.0% | 50.0% |
| 5-May-12 | 3 | 3 | 2 | 2 | 66.7% | 66.7% |
| 6-May-12 | 3 | 3 | 3 | 2 | 100.0% | 66.7% |
| 7-May-12 | 2 | 2 | 0 | 0 | 0.0% | 0.0% |
| 8-May-12 | 3 | 3 | 0 | 2 | 0.0% | 66.7% |
| 9-May-12 | 3 | 3 | 0 | 0 | 0.0% | 0.0% |
| 10-May-12 | 7 | 5 | 0 | 0 | 0.0% | 0.0% |
| 11-May-12 | 4 | 3 | 4 | 1 | 100.0% | 33.3% |
| 12-May-12 | 7 | 3 | 3 | 0 | 42.9% | 0.0% |
| 14-May-12 | 3 | 2 | 2 | 2 | 66.7% | 100.0% |
| 15-May-12 | 4 | 2 | 0 | 1 | 0.0% | 50.0% |
| 16-May-12 | 4 | 2 | 0 | 0 | 0.0% | 0.0% |
| 17-May-12 | 8 | 2 | 0 | 0 | 0.0% | 0.0% |
| 18-May-12 | 4 | 2 | 0 | 0 | 0.0% | 0.0% |
| 22-May-12 | 4 | 3 | 0 | 0 | 0.0% | 0.0% |
| 30-May-12 | 4 | 1 | 0 | 0 | 0.0% | 0.0% |
| 18-Jun-12 | 8 | 6 | 1 | 2 | 12.5% | 33.3% |
