# Supplementary material for: Correction: Year-Round Reproduction and Induced Spawning of Chinese Amphioxus, Branchiostoma belcheri, in Laboratory
Source: PLoS One. 2014 May 27;9(5):e99264. doi: 10.1371/journal.pone.0099264 (PMC4035332; doi:10.1371/journal.pone.0099264)
Supplement: File S1 — Originally published, uncorrected article. (PDF) [file pone.0099264.s001.pdf]

Year-Round Reproduction and Induced Spawning of Chinese Amphioxus, *Branchiostoma belcheri*, in Laboratory

Guang Li[1,2], ZongHuang Shu[1], Yiquan Wang[1,2*]

1 School of Life Sciences, Xiamen University, Xiamen, China, **2** Shenzhen Research Institute of Xiamen University, Shenzhen, China

Abstract

Amphioxus is a best candidate for studying the evolutionary and developmental mechanisms of vertebrates, because of its vertebrate-like but much simpler morphology, embryonic development and genome structure. Producing live amphioxus embryos throughout the year is an ideal for comparative evolution and developmental studies. However, all amphioxus species have distinct breeding seasons in the wild and laboratory. We recently found that Chinese amphioxus *B. belcheri* could reproduce repeatedly beyond its natural breeding season when reared under proper conditions. In this study, we were able to extend further and produce embryos throughout the year from October 2011 to October 2012. We found all examined animals had spawned repeatedly during the examined period. In addition, both lancelets *B. belcheri* and *B. japonicum* could be induced to spawn by heat-shock method, although the induced spawning efficiency was not as high as that observed in the European lancelet. In general, we have succeeded in producing *B. belcheri* embryos almost daily throughout the year. This advancement will provide essential embryonic material for evolutionary and developmental studies, and have great implications for the cultivation and spawning induction of other amphioxus species.

Citation: Li G, Shu Z, Wang Y (2013) Year-Round Reproduction and Induced Spawning of Chinese Amphioxus, *Branchiostoma belcheri*, in Laboratory. PLoS ONE 8(9): e75461. doi:10.1371/journal.pone.0075461

Editor: Hector Escriva, Laboratoire Arago, France

Received February 23, 2013; **Accepted** August 15, 2013; **Published** September 26, 2013

Copyright: © 2013 Li et al. This is an open-access article distributed under the terms of the Creative Commons Attribution License, which permits unrestricted use, distribution, and reproduction in any medium, provided the original author and source are credited.

Funding: This work was supported by the Natural Science Foundation of Fujian Province of China (No. 2011J05097), the National High Technology Research and Development Program of China (No. 2008AA092602), the National Natural Science Foundation of China (No. 30830023 and No. 31101631) and Social Development and Technological Project by the Department of Science and Technology of Shenzhen (No. JSF201006290026A). The funders had no role in study design, data collection and analysis, decision to publish, or preparation of the manuscript.

Competing interests: The authors have declared that no competing interests exist.

* E-mail: wangyq@xmu.edu.cn

Introduction

Phylum Chordata consists of three subphyla: Cephalochordata (commonly called amphioxus or lancelets), Urochordata (also known as tunicates) and Vertebrata. From the views of morphology, development [1] and small sets of molecular data [2], Cepholachordata used to be classified as a sister group of Vertebrata, whereas Urochordata positioned at the base of the phylum. However, several recent studies based on large molecular datasets demonstrated that cephalochordates diverged earlier than tunicates during the evolution of chordates [3-6]. This revised phylogeny, therefore, makes the cephalochordate amphioxus the best available proxy for understanding the genetic basis of chordate development. Among the two invertebrate chordate subphyla, tunicates have evolved extensively since the divergence from vertebrate lineage, but cephalochordates appear to maintain most features of their chordate ancestors. For example, amphioxus possesses a dorsal hollow nerve cord, notochord, segmental muscles throughout its life cycle, but tunicate has only these tissues/organs transiently in the embryonic stages [1]; amphioxus retains numerous ancestral chordate genes, non-coding regulatory elements and genomic structure, but tunicate lacks most of these contents due to extensive gene loss and genome rearrangements [6-10]. Most importantly, amphioxus is much simpler in embryonic development, body structure as well as genome contents compared to the complexity of vertebrates. Amphioxus embryos show little cell involution during gastrulation, and lack definitive neural crest cells, placodes and complex brain structures [11]; its genome has not underwent extensive genome duplications [6,12]. Because of these advantages, amphioxus has been considered as an ideal model animal for studying vertebrate evolution and development [13-16].

Until now, amphioxus has not yet become a wide-use laboratorial animal mainly due to the limited and unpredictable supply of embryos. Following success in continuous culture and inbreeding of amphioxus in our laboratories [17,18], we sought a reliable laboratory culture system and methods that could produce live embryos daily throughout the year. Raising

ripe animals in non-breeding season and developing an efficient spawning-induction method are two prerequisites to reach above aim. Currently, four amphioxus species, *Branchiostoma floridae*, *B. lanceolatum, B. belcheri* and *B. japonicum* have been commonly used for developmental studies [15,16,19-21]. All of these species have distinct breeding seasons varying from a couple of weeks (for *B. japonicum*) to several months (*B. lanceolatum*, *B. floridae* and *B. belcheri*) [20,22,23]. Except *B. lanceolatum*, which could be induced to spawn by heat-shock in the breeding season [23-25], the other three species only spawn on unpredicted dates during the breeding season [15]. We recently found that Chinese amphioxus *B. belcheri* could spawn consecutively in captivity extend beyond its breeding seasons [26]. This finding indicates possible gonadal recrudescence in *B. belcheri* throughout the year. In this study, we extended our observation on the spawning behavior of *B. belcheri* and found that majority of examined individuals could spawn repeatedly, with some individuals spawning up to eight times. We applied this method on large-scale cultivation of *B. belcheri* and obtained plenty of mature animals since the 2011 breeding season. In addition, we also showed that both *B. belcheri* and *B. japonicum* could be induced to spawn by temperature shifting, although the induced spawning efficiencies are not as high as that of *B. lanceoletum* [23-25]. We provided a detailed description about the induced spawnings of *B. belcheri* from June 2011 to October 2012, and of *B. japonicum* in the 2011 and 2012 breeding seasons.

Materials and Methods

Animal source used in the study

Amphioxus adults (*B. belcheri* and *B. japonicum*) were collected several times from the field in 2008 in order to establish a few breeding populations in our laboratory. Following each collection, *B. belcheri* and *B. japonicum* animals were identified according to the previous descriptions [20,21,27], and cultured separately. Approximate ten thousands of wild *B. belcheri* and *B. japonicum* adults were collected. Thereafter, amphioxus (~150 per collection) was occasionally sampled from the field in order to monitor their gonadal recrudescence for a reference to the laboratory counterparts. Benefit from the realization of continuous culture of the two lancelets [18], several thousands of offspring were generated and preserved in our lab every year. When these offspring became sexually mature they were mixed together with their parents and used in the present study.

Animal culture conditions in general

All animals used in the present study were raised in natural seawater. Generally, adult *B. japonicum* were cultured under natural photoperiod and room temperature in the non-breeding season according to Zhang's description [18], but were subjected to a 13.5/10.5 hour day/night cycle (00:00-13:30 and 13:30-00:00) at 19°C (water temperature) in the breeding season. Adult *B. belcheri* were maintained under similar conditions before October 2011, but after that, if not otherwise stated, they were cultured using a modified procedure described recently [26]. This revised procedure differed from Zhang's description mainly in: 1) the stocking density was decreased from about 1600 individuals to 550 individuals per square meter as described in the following sections; 2) the water temperature was maintained between 25°C to 28°C for animals with no obvious gonads or small gonads, and 19°C or 22°C for animals with medium or large gonads; 3) the animals were exposed to a 13.5-hr light/10.5-hr dark cycle in which the light period was started at 00: 00 PM and ended at 1: 30 PM of next day; 4) the animals were fed twice a day with mixed fresh algae plus commercial shrimp flakes (Sailboat Brand, produced by Bonasse Biochemistry Technology Enterprise Co., Ltd, Taipei) at 9:00-10:00 and 17:30-18:00 respectively, and cleaned every 10-15 days. Animals with medium or large gonads were defined by two criteria: 1) the horizontal width of most of their gonads is greater than 0.8 mm, and 2) the gonads are filled with cloudy or thick gametes. For a straightforward example, please see pictures Figure 3 in our previous study [26]. Those pictures not marked with red arrows indicate animals with medium or large gonads.

Small-scale culture for assessing the consecutive spawning of *B. belcheri*

The animals examined in this experiment were the same cohort used in the Experiment I of our previous study [26]. They were maintained at 25°C to 28°C and subjected to a 13.5/10.5 hour day/night cycle (00:00-13:30 and 13:30-00:00). These animals were divided into two groups: Group I included 24 females and 24 males spawned on October 30th, 2011 and Group II included 22 females and 43 males spawned on November 14th, 2011. We previously reported our results on their consecutive spawning behaviors till April 28th, 2012 [26]. To determine whether they could spawn repeatedly throughout the year, we continued our observation till October 2012, about one year after the experiment started in October 2011. Animal rearing and data collecting methods were the same as our previous report [26]. Briefly, the animals were reared in substrate sand in 5 liter red plastic barrels (less than 25 individuals per barrel) and fed with mixed fresh algae three times a day. The substrate sand and containers were cleaned once a week to keep a clean living environment. At beginning of the experiments, animals were named "First Spawning" animals. The development of gonads was visually examined once every ten to fifteen days because of their transparent bodies and visible gonads. After each examination, animals with small or spent gonads were counted and transferred into 5 liter red plastic barrel(s), and those with medium or large recrudescent gonads were transferred into other 5 liter red plastic barrels. These animals with recrudescing gonads were called "Second Development" animals. Their subsequent spawning was also examined visually every ten to fifteen days, supplemented with a daily check in the afternoon. Once animal released gametes, we would found animals with small or spent gonads (not visible) again, and therefore, we define the spawning as "Second Spawning", and these kinds of animals entered "Third Development" stage. These spawned animals were counted and transferred into different 5 liter red plastic barrel(s). Consequently, we have "Third Spawning" and "Forth

Development" animals and so on. In addition, we introduced two other parameters to evaluate animals, gonad recrudescence ratio (GRR) and spawning ratio (SR). GRR represents the number of animals that have re-filled their gonads after spawning within a month over the total number of animals, and SR is the number of animals that have spawned after their gonads were re-filled in a month to the total number of animals.

Large-scale culture for obtaining large number of mature *B. belcheri* individuals

In order to examine whether large numbers of mature animals could be obtained throughout the year, we scaled up our protocol for a large-scale cultivation started from October 2011. For this purpose, we collected about 3,000 adult amphioxus, randomly distributed about 60 individuals per custom-made acrylic tank (400mm×260mm×190mm) filled with 8 liter seawater and substrate sand. These tanks were numbered and organized on steel selves. Every 15 to 20 days, we screened out the animals and selected individuals with mature gonads for subsequent spawning induction.

Temperature induced spawning in *B. belcheri* and *B. japonicum*

A thermal shock method, modified from an updated protocol for *B. lanceolatem* [25], was used for spawning induction of *B. japonicum* and *B. belcheri*. This method included three steps: 1) collect the mature animals as described above, and maintain them at a low temperature for a minimal of five days (the temperature was set at 19°C for *B. japonicum* and 19°C or 22°C for *B. belcheri*); 2) at about 10 AM on day n, transfer some of mature animals from 'maintaining tanks' (large tanks) to 'spawning tanks' (small ones) at a high temperature with sand and continuous aeration (24°C for *B. japonicum* and 27°C for *B. belcheri*), and feed the animal with mixed fresh algae and commercial shrimp flakes; 3) at about 11 AM on day n+1, transfer the animals into individual plastic cups with approximately 20 mL of filtered warm seawater, and seat the cups back into the warm water bath. Spawning was examined every 30 minutes till 17:30 after light was off at 13: 30. Animals that did not spawn after heat shock were transferred back to regular tanks for spontaneous spawning and would not be used for further inducing experiments. Animals that were successfully induced to spawn were transferred back to the regular tanks for gonadal recrudescence.

Results

Year-round consecutive spawning of *B. belcheri* in captivity

Recently, we reported the consecutive spawning of *B. belcheri* adults from October 2011 to April 2012 [26]. To assess whether the adults could spawn repeatedly throughout the year, we extended our observations on the same set of animal to a year round from October 2011 to November 2012. During this period, number of animals decreased from 49 to 45 in Group I and from 65 to 49 in Group II, caused mainly by animal death or occasionally loss during data collecting process. All remaining animals reproduced consecutively throughout the year (Figures 1 & 2). About 84% (38/45) of animals in Group I spawned at least five times (Fifth Spawning; Figure 1) during a 362-day experimental period. Six individuals entered the 8th development, while 2 individuals spawned 8 times (the 8th Spawning) (Figure 1). In Group II, thirty of 49 examined animals (67%) spawned at least four times during a 289-day experimental period. However, three individuals in Group II had a relatively long interval between their spawnings (highlighted in a thin black box in Figure 2). To simplify, these three animals together with the other 13 individuals in early stages (highlighted in a thick black box in Figure 2) were excluded from subsequent analyses. On the 10th November 2012 (363 days after the experiment started), all the remaining 33 animals in Group II entered into the 6th Development or late (Figure 2).

To further assess if recurring spawning in *B. belcheri* varies among different seasons, we determined the percentages of animals with recrudescent gonads (GRR) and newly spent gonads (SR) every month. As is shown in the inserts of Figure 1 and Figure 2, GRR varied from 0 to 56% (average= 36%) in Group I and 19% to 74% (average= 44%) in Group II; and SR varied from 4% to 78% (average= 41%) in Group I and 2% to 80% (average= 40%) in Group II. Two-tailed chi-squared test using the Crosstabs procedure in SPSS software indicated that both GRR and SR varied significantly among different months in both groups (P = 1.24×10^{-10} and P = 1.03×10^{-24} for Group I; P = 4.74×10^{-7} and P = 4. 47×10^{-41} for Group II). Unexpectedly, we found that animals in Group I exhibited relatively low SRs in July and August, part of the natural breeding season of the species (June to September). This result suggests that the spawning of the animals, which were kept under a constant temperature and photoperiod (see Materials and Methods), has a different reproductive cycle from those in the wild. In addition, under the conditions, we were able to obtain spawning animals in all examined twelve months despite a small size in both groups (~50 individuals). The result suggests that we can simply increase the number of the animals in lab to obtain sufficient mature animals frequently throughout the year. This idea has been tested and proved to be practicable in our laboratory since October 2011. About three thousand of *B. belcheri* adults, which were produced in our lab or collected from field in 2008, were cultured under similar conditions as described in the small-scale cultivation (see Materials and Methods). More than 100 individuals with medium or large gonads could be selected from the population every 15 to 30 days, which provided sufficient mature animals for spawning induction and other routine experiments in our laboratory.

To examine whether *B. japonicum* could reproduce repeatedly throughout the year, we selected 270 similar-sized animals and divided them into three groups. Each of them was reared under similar conditions as described in *B. belcheri* but at three different temperatures (18-19°C, room temperature, and 25-27°C respectively) under natural photoperiod for over one year (24th Nov. 2011 to 22nd Mar. 2013). About 50.0% (42/84, reared at 25-27°C), and 18.8% (16/85; reared at room temperature) of animals spawned twice during the breeding season from February to May in 2012, but all animals reared at

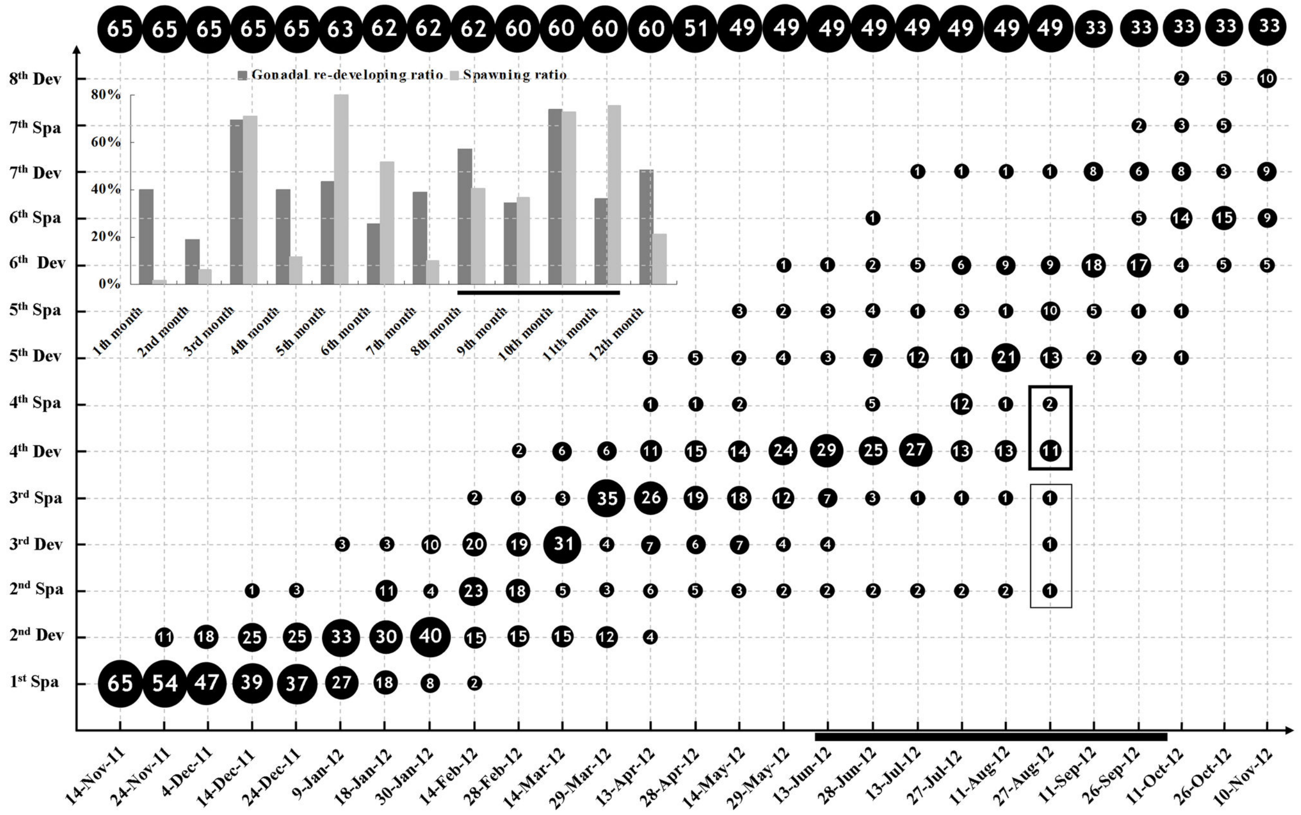

Figure 1. Gonadal recrudescence and spawning of *B. belcheri* in Group I. Numbers of animals with different reproductive phases on each examined day are shown in the diagram roughly in scale. Numbers at the top of the diagram are total numbers of animals on each examined day. Percentages of gonadal recrudescence and spawned animals in each month are shown in the insert bar graph. Gonadal recrudescence ratio (GRR) and spawning ratio (SR) respectively represents the number of animals with recrudescing gonads and the number of animals spawned over the number of total animals examined in each month. Dates corresponding to the natural breeding season (June to September) are marked by bold horizontal lines. Abbreviations: Dev, Development; Spa, Spawning.

doi: 10.1371/journal.pone.0075461.g002

18-19°C spawned only once during the period. No spawning was observed for all three groups of animals in non-breeding season (see Table S1 for detail).

Temperature induced spawning in *B. belcheri*

A thermo-based spawning induction method developed in *B. lanceolatum* [23-25] was modified to stimulate spawning of ripe *B. belcheri*. Two different temperature shifts (19°C to 27°C and 22°C to 27°C) were tested for a high induced spawning efficiency. During the period between 7th June 2011 and 12th April 2012, we maintained the ripe amphioxus at 19°C and stimulated them for spawning at 27°C (Method I). After that (between 12th April 2012 and 23rd October 2012), we increased the maintaining temperature to approximate 22°C and still used 27°C to induce the spawning (Method II). The percentage of spawning males (in blue) and females (in red) is plotted against each induced spawning dates (Figure 3). Induced spawning in both sexes on a given date did not begin until 25th July 2011, which is thus defined as the beginning of the breeding season for *B. belcheri* in 2011. Thereafter, we conducted spawning induction experiments on 144 separate dates using Method I, and on 98 separate dates using Method II. Method I induced animals were further divided into two groups. One group was cultured using Zhang's conditions from July to October in 2011, and the another group using our updated protocol from November 2011 to the beginning of April 2012. We therefore analyzed the recording data separately. Under Zhang's raring conditions and Method I (74 inductions were conducted), 20 inductions (27.0%) led spawning of both males and females, 8 inductions (10.8%) of males only, 5 inductions (6.8%) of females only, and 41 inductions (55.4%) failed to lead spawning (Figure 3a). Using our updated raring conditions and Method I (70 inductions were conducted), 24 inductions (34.3.0%) led spawning of both males and females, 8 inductions (11.4%) of males only, 9 inductions (12.9%) of females only, and 29 inductions (41.4%) failed to lead spawning (Figure 3a). Among the 98 induction experiments using Method II, 51 inductions (52.0%) led spawning of both males and females, 8 inductions (8.2%) of males only, 21

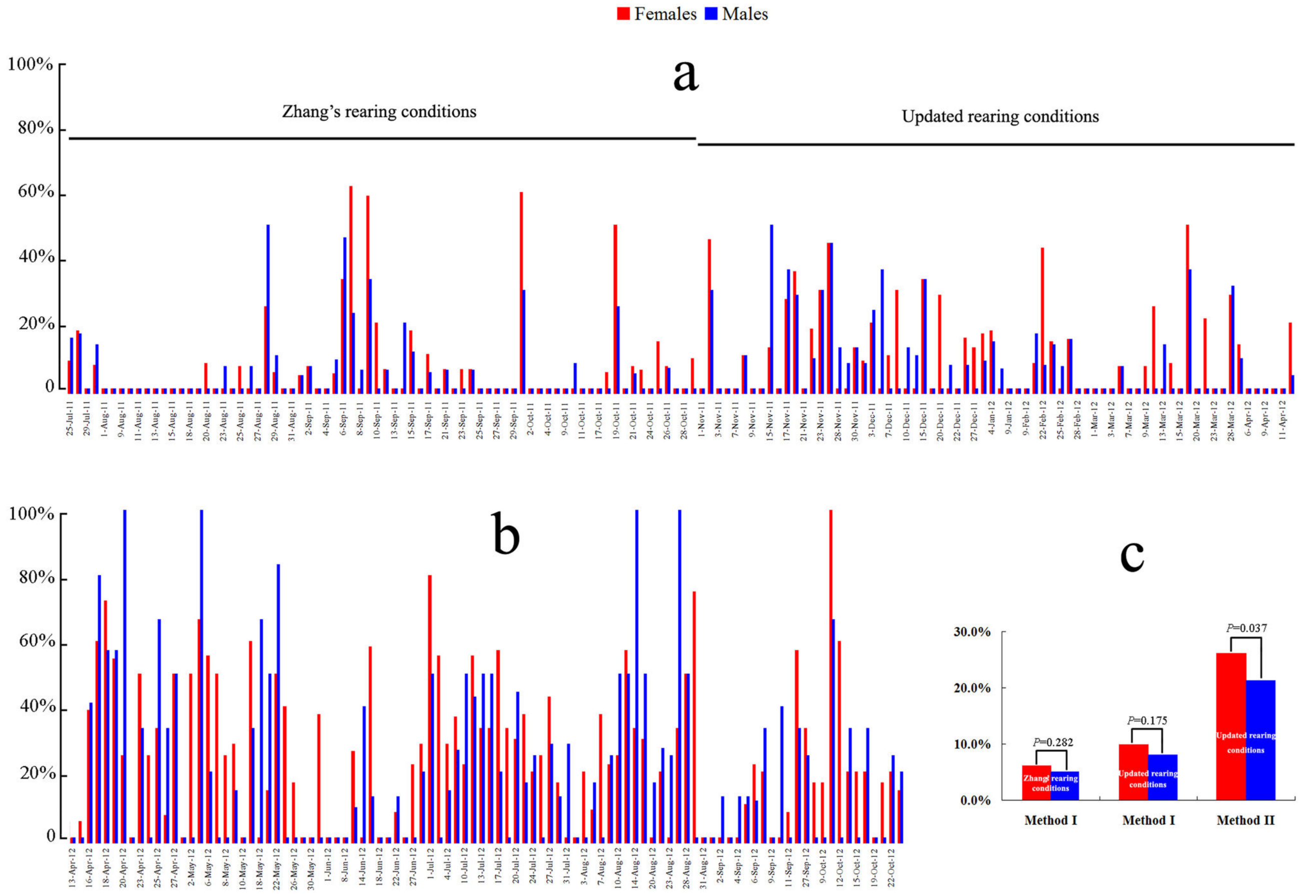

Figure 2. Recurring gonadal recrudescence and spawning of *B. belcheri* in Group II. Numbers of animals with different reproductive phases on each examined day are shown in the diagram roughly in scale. Numbers at the top of the diagram are total numbers of animals on each examined day. Animals in the black boxes were excluded from the subsequent analysis due to their relatively low rates of gonadal development. Percentages of gonadal recrudescence and spawned animals in each month are shown in the insert. Gonadal recrudescence ratio (GRR) and spawning ratio (SP) represents respectively the number of animals with recrudescing gonads and the number of animals spawned over the number of total animals in each month. Dates corresponding to the natural breeding season (June to September) are marked by bold horizontal lines. Abbreviations: Dev, Development; Spa, Spawning.

doi: 10.1371/journal.pone.0075461.g003

inductions (21.4%) of males only, and 18 inductions (18.4%) did not induce any spawning of both males and females (Figure 3b). The cumulatively induced spawning efficiencies for the two sets of animals subjected to the Method I are 5.1% and 6.1% for males and 8.0% and 9.9% for females, and that for animals applied Method II is 21.2% for males and 26.1% respectively (Figure 3c). Statistical analysis indicated that the spawning efficiency between males and females was significantly different in Method II (P = 0.037, 2-tailed chi-squared test), but not in Method I (P=0.282 and P=0.175 for the two rearing conditions, two-tailed chi-squared test) (Figure 3c). To further test whether there is a statistical difference for the induced spawning efficiency between Method I an II, we conducted a parallel spawning induction experiments using two different temperature shifts. Two cohorts of animals (about 100 individuals each) with medium or large gonads were selected respectively on 26th March and 1st April from the population that were cultured under our updated method. Method II showed higher mean induced spawning ratio than Method I in both male and female *B. belcheri* (17.6% *vs.* 11.6% for males and 26.3% *vs.* 10.5% for females; Figure S1). However, statistical analysis only detected a week significant difference between two methods in females (P = 0.069, one-tailed fisher's exact test), but no significant difference in males (P= 0.335).

We next analyzed whether the efficiency of induced spawning varies among different months. For each month, the percentage of induced spawning dates was employed to assess the induced efficiency in a given month. From July 2011 to October 2012, total of 252 inductions were performed, with 4 to 26 (average= 14.8) inductions per month. The induced efficiency among different months varied from 0 to 26.7% (average= 11.3%) in males only, 0 to 40% (average= 13.1%) in

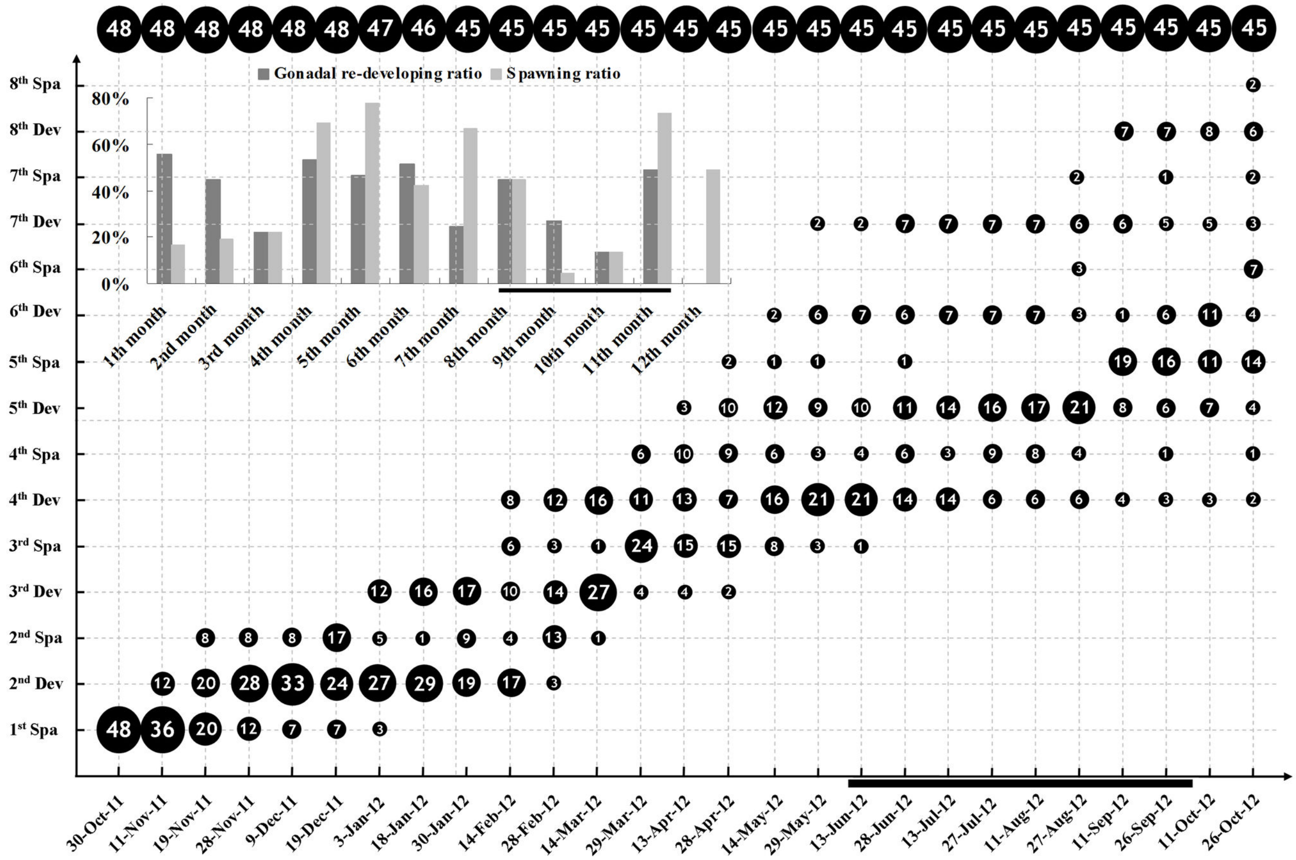

Figure 3. Year-round spawning inductions in *B. belcheri* from 25th July 2011 to 23rd October 2012. Percentage is the number of spawned animals (males or females count separately) over the total number of temperature shocked males or females in each examined date. Inductions using temperature shift from 19°C to 27°C (Method I, from 25th July 2011 to 12th April 2012) and from 22°C to 27°C (Method II, from 13th April to 23rd October 2012) are shown in the figures **a** and **b**. The Figure **c** shows the cumulative spawning percentages of males and females using Method I or II. Statistical analyses of the data were carried out using SPASS software (version 16.0) and two-tailed chi-squared test. All raw induction records are listed in Table S3.

doi: 10.1371/journal.pone.0075461.g001

females only, and 8.7% to 76.5% (average= 36.4%) in both males and females (Figure 4). Percentages of the dates in which both males and females spawn were significantly different among different months (P = 0.004) as analyzed by two-tailed chi-squared test using the Cross tabs procedure in SPSS software. Under our rearing conditions, the efficiency of temperature induced spawning in *B. belcheri* reached a peak within two or three months, which had no correlation with the natural breeding season (from June to September). It is important to note that we successfully induced spawning in both males and females on variety days in each of the sixteen examined months.

By assaying the presence of animals with empty gonads from 12th April to 23rd October 2012, we found that nearly half (45%) of animals spawn spontaneously prior to thermal shock (Figure 5a). A similar phenomenon was also observed in animals maintained at about 19°C (data not shown). Side-by-side experiments indicated that animals kept at 22°C tended to spawn spontaneously than those kept at 19°C (Table S2). In addition, for the same sets of animals, we also monitored their spawning dynamics. About 15% of spawning in both sexes occurred within 30 minutes following light off, and 50% of them within 0.5-1.5 hr. Only 3% males and 5% females spawned after 2.5 hr in the dark (Figure 5b).

Temperature induced spawning in *B. japonicum*

We also studied *B. japonicum* cultured in our laboratory to find out whether temperature induced spawning works in a different lancelet species. *B. japonicum* mainly distributes along the north coast of West Pacific ocean [28] and belongs to temperate lancelets like the European amphioxus *B. lanceolatum*. Therefore, we used a temperature shift (from 19°C to 24°C) similar to the temperature shift (from 19°C to 23°C) used in *B. lanceolatum* [23-25] for spawning induction of *B. japonicum*. Following the thermal shock, males and females began to spawn on 10th March and ended on 10th May in 2011 (Figure 6). During this period, total of 37 inductions were conducted. Among these inductions, spawning of both sexes

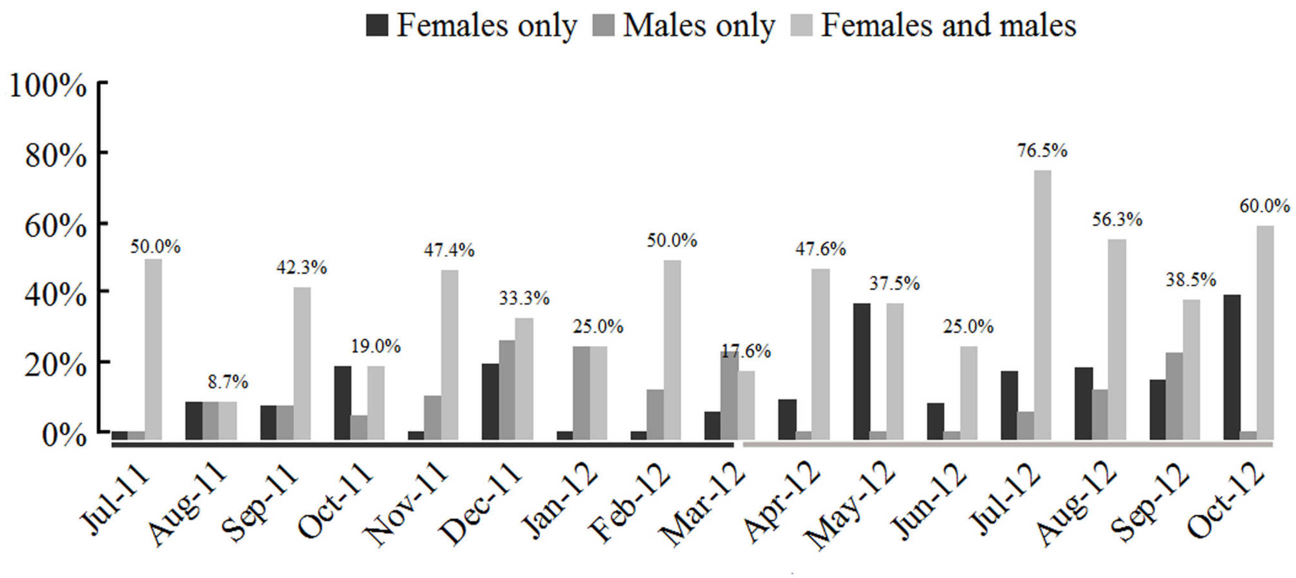

Figure 4. Percentage of induced spawning dates of *B. belcheri* for each of 12 examined months. For each month, the spawning percentage is the number of spawning dates (males only, females only, or both sexes) over the total number of dates on which the spawning induction are conducted. The periods used the two-shock systems are respectively highlighted by black and grey horizontal bars. Natural breeding of *B. belcheri* occurs from June to September.

doi: 10.1371/journal.pone.0075461.g004

was observed in 28 inductions (73.7%), males spawning only were observed in 9 (26.3%) inductions, whereas none of female only spawning was recorded. The overall induced spawning efficiency was significantly different in males *vs.* females (31.2% *vs.* 13.7%, P = 4.36×10^{-13}, two-tailed chi-squared test). In 2012, both sexes were able to be induced to spawn from 7th April to 18th June, about one month later than those in 2011. During the period, we conducted thermal inductions in 38 days. Among these inductions, both sexes spawned in 21 inductions (55.3%), male only spawning were observed in 3 (7.9%) inductions, female only spawning were observed in 5 (13.2%) inductions, whereas no spawning were observed in 9 (23.7%) inductions. Again the cumulative induced spawning efficiency was significantly higher in males than that in females (47.9% *vs.* 29.5%, P = 0.001, two-tailed chi-squared test). Interestingly, the overall induced spawning efficiencies of both sexes were higher in 2012 than that in 2011, whereas the percentage of the dates in which both sexes spawned in 2012 was lower than that in 2011. The difference was probably due to increased number of animals used in most of the inductions in 2011 than that in 2012. Average males and females used for each induction were 12 ± 6 and 20 ± 7 in 2011, compared to 4 ± 3 and 6 ± 3 used in 2012. The duration of induced spawning in 2011 and 2012 lasted about two months, which exceeded the natural spawning season of *B. japonicum* (from late April to early May) [20] and *B. lanceolatum* [23]. Like *B. lanceolatum*, male *B. japonicum* generally mature and spawn earlier than females (data not shown). Spawning typically reached peak around 2 hours following the lights off, and most of the animals completed spawning within 2.5 hours after dark (Figure 5c).

Discussion

Amphioxus has long been considered as a promising model organism; however, several crucial shortcomings need to be overcome before amphioxus becomes an ideal laboratorial model animal. Among these limitations, a reliable laboratory culture system allows daily supply of freshly spawned gametes is a key for developing molecular tools, establishing embryonic manipulation techniques and conducting routine laboratory experiments. Aiming to establish such system, here we described environmental conditions for maintaining mature *B. belcheri* throughout the year and a temperature-shift spawning method for daily requirement in the laboratory. Besides, we also established a similar temperature shift method for inducing spawning in *B. japonicum* in 2011 and 2012 breeding seasons. In the lab, we had an over 50% success rate in obtaining freshly released amphioxus gametes. Our study also suggests that year-round cultivation and spawning induction might also be possible for other commonly used amphioxus.

Year-round spawning in *B. belcheri*

In our previous study [26], we demonstrated that adult amphioxus *B. belcheri* could spawn consecutively during and beyond the natural breeding season under appropriate conditions. In current study, we continued one of the three experiments set in the previous study for over one year. We found all examined animals could keep this consecutive spawning throughout the year. However, the spawning frequency varies considerably between different individuals. Among the 45 individuals examined in Group I, two spawned as many as seven times (not include the first spawning when

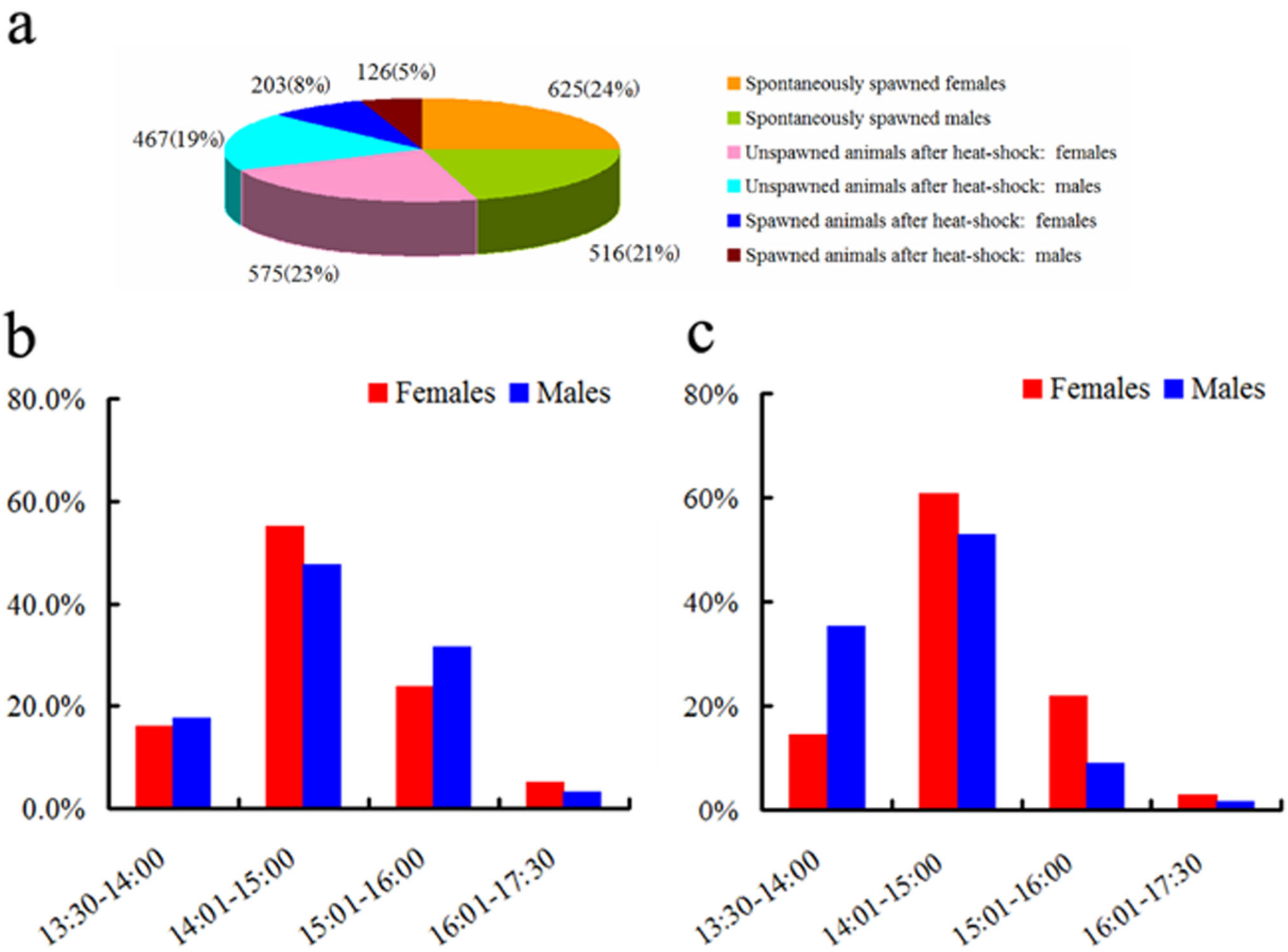

Figure 5. Percentages of spawned animals and spawning time in *B. belcheri* and *B. japonicum*. a. The cumulative percentages of the spontaneously spawned, induced spawned and not spawned animals in *B. becheri* during the period from 13th April to 23rd October 2012 when Method II shock system was used. There are three different outcomes for mature animals selected and kept at 22°C: 1) those spontaneously spawned prior to heat shock (in 22°C water both), 2) those spawned after heat shock, and 3) those did not spawn even after heat shock. We calculated percentages separately according to sex. For each group of animals, their numbers and ratios (in the bracket) are shown. b. Spawning time and percentages of spawned male or female *B. belcheri* following light off at 13:30. Only the data using Method II (from 13th April to 23rd October 2012) is shown. c. Spawning time and percentages of spawned male or female *B. japonicum* following light off at 13:30. Only the data in the 2012 breeding season is shown.

doi: 10.1371/journal.pone.0075461.g005

the experiment was started), but another two spawned just three times (Figure 1). A similar result was also observed in Group II (Figure 2). This variation might be due to the high genetic diversity of amphioxus population and their varied responses to environmental cues [26]. Our observation also revealed that a small breeding population (approximately 50 individuals) is sufficient for maintaining mature individuals and obtaining mature gametes in our lab throughout the year. In our large culture (~3000 adults), 100 to 200 animals with medium or large gonads could be selected from the population every 15 to 30 days. This provides us sufficient mature animals for spawning induction and large number of live embryos for the routine laboratory experiments.

Implications of year-round spawning in other lancelet species

Four species from genus *Branchiostoma* are commonly used for developmental studies. All of these species have distinct breeding season in the field [16,26]. Among them, *B. japonicum* and *B. lanceolatum* belong to temperate lancelets, while *B. belcheri* and *B. floridae* are subtropical species [22]. Early studies suggested that *B. japonicum* shared more similarities with *B. lanceolatum* in the spawning behaviors, whereas *B. belcheri* was closer to *B. floridae* (as discussed in 26). This assumption was further supported by the finding in the present study that *B. japonicum* and *B. lanceolatum* appear more sensitive to heat shock and generally show a higher

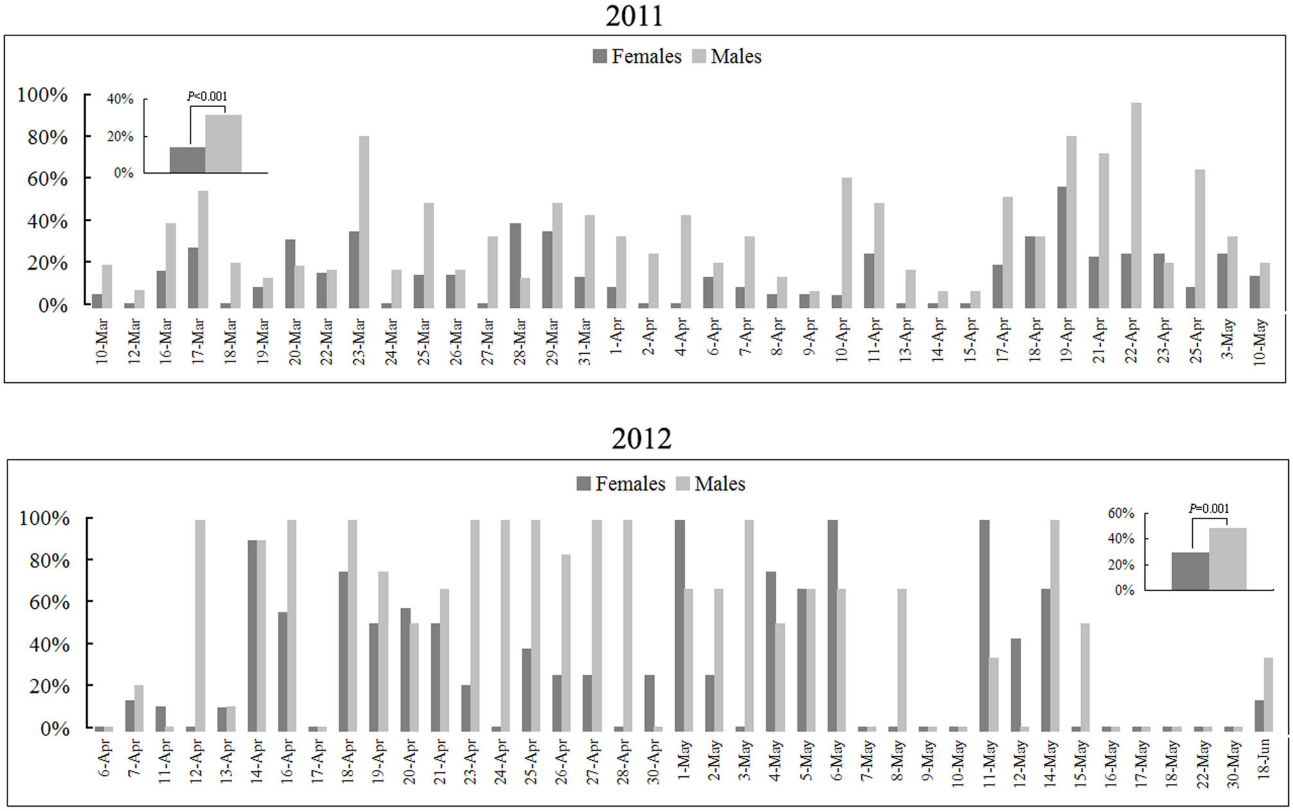

Figure 6. Percentages of induced spawning in *B. japonicum* during 2011 and 2012 breeding seasons. For each examined date, the spawning percentage is the number of spawned animals (males and females, respectively) over the number of shocked animals (males and females, respectively). The insets show the cumulative spawning percentages of males and females in each of the two breeding seasons. Statistical analyses of spawning efficiency between shocked males and females were carried out using SPASS software (version 16.0) and two-tailed chi-squared test. All raw induction records are listed in Table S4.

doi: 10.1371/journal.pone.0075461.g006

efficiency of induced spawning than that of *B. belcheri* (see the discussion below). Therefore, we expect that *B. floridae* may also spawn consecutively all the year round in lab as we found in *B. belcheri*. As for *B. japonicum* in our experiment, we did not find obvious signs indicating the repetitively spawning throughout the year. But we noticed that adult *B. japonicum* could advance their breeding season (data not shown) 2 months earlier when raised at high temperatures. This result, together with the finding that the unseasonable cool water retarded the onset of *B. japonicum* breeding season [29], suggested that year-round ripe *B. japonicum* (or *B. lanceolatum*) individuals might be obtained by keeping different sets of animals for breeding in different seasons, altering the water temperature and ambient light, and by feeding or stocking appropriately.

Thermal shock induced spawning in lancelets

The thermal shock method was first developed for the spawning induction in *B. laceolatum* in 2004. Since then, the method has been repeatedly used in this species in the breeding season [23-25]. So far, no such methods have been successfully developed in other lancelet species. Ripe *B. floridae* adults were able to be induced to spawn by electroshock, but only on the dates when they spawned in the field [30]. This electroshock method is unpredictable and could not provide embryonic materials to meet the demands. In the present study, we have successfully established the thermo-based spawning induction method in both *B. belcheri* and *B. japonicum*. Our result strongly suggests that the thermo-based method is a universal method for spawning induction in lancelets.

The temperature shift used for the spawning induction in *B. lanceolatum* animals is from 19°C to 23°C [23-25]. Considering of *B. japonicum* is a temperate lancelet like *B. lanceolatum*, we thus adopted a similar temperature shift (from 19°C to 24°C) for spawning induction in *B. japonicum*. The efficiencies of induced spawning in *B. japonicum* were 31.2% in males and 13.7% in females in 2011, and 47.9% in males and 29.5% in females in 2012 (Figure 6), which were comparable to those (around 30% for both sexes) found in *B. lanceolatum* described recently [25]. As for the sub-tropical species *B. belcheri*, we initially used a 19°C-to-27°C temperature shift (Method I) to induce their spawning and only observed a relatively low efficiency in

spawning induction in both sexes. We found that *B. belcheri* did not eat very well at 19°C. Considering of the water temperature at the onset of the breeding season of *B. belcheri* was about 22°C [18], we speculated that 19°C might not be good for the gonadal maturation. We therefore increased the maintaining temperature to 22°C and still used 27°C temperature to induce spawning (Method II). Method II was more efficient than Method I for the spawning induction of *B. belcheri* adults in our hands. This conclusion was further supported by experiments using two methods in parallel. However, it should be noted that animals kept at 22°C seemed to have more spontaneous spawning than those at 19°C.

The efficiency of induced spawning in male *B. japonicum* appeared to be significantly higher than that in females (Figure 5c). A similar result was also observed in repeated shocked *B. lanceolatum* animals but not in the first shock [25], and in *B. belcheri* when the Method II was used (Figure 3c). We found that some individuals of both *B. japonicum* and *B. belcheri* would spontaneously spawn even when they were kept under a constant low temperature, which is similar to that in *B. lanceolatum* [25]. This result indicated that unknown environmental and/or internal factors, could also trigger the spawning of lancelets [23]. After the heat shock, the induced spawning in male *B. japonicum* appeared to be earlier than that in females (Figure 5c). This observation is similar to that observed in *B. lanceolatum* [25], but was not obvious in *B. belcheri* (Figure 5b). Among these three species, spawning in both sexes reached peak around 2 hr following the light off (Figure 5b and c here, and Figure 6 in the reference [25]). Over 95% of both *B. belcheri* and *B. japonicum* males and females completed their spawning within the 2.5 hr after dark (Figure 5b and c). Only a small fraction of *B. lanceolatum* (including both males and females) could spawn even 5 hr after dark [25].

Factors affect the efficiency in temperature-shift induced spawning

Success in temperature-shift induced spawning in three lancelet species provides an opportunity to seek out factors that could affect the efficiency of induced spawning. Our current results and the previous reports [23-25] indicate three factors seem to be crucial for successful spawning induction in amphioxus. The first factor is an internal determinant. As we discussed above, the temperate lancelets seem to be more sensitive to thermal shock than the sub-tropical ones, and thus show higher efficiencies in induced spawning in both sexes. Secondly, optimal temperatures for maintaining and shocking the ripe animals also appear to be very important. A proper maintaining temperature should mirror to natural temperature when lancelets develop and ripen their gonads in the sea; and shocking temperature should approach to that when animals spawn spontaneously in the field. Both *B. lanceolatum* and *B. japonicum* develop and ripen their gonads at approximately 10-19°C and spawn when the temperature is above 23°C in the field [20,23]. So, high efficiencies of induced spawning were observed in both species when the temperature shifts from 19 to 23 or 24°C (Figure 6 in this study and Figure 4 in the reference [25]). In contrast, *B. belcheri* animals need a relatively higher temperature (about 22-26°C) to develop and release their gametes [20]. We observed a higher efficiency of temperature induced spawning using Method II (shift from 22°C to 27°C) than those using Method I (shift from 19°C to 27°C) (Figure 3). However, we noticed that more mature animals maintained at 22°C tend to spawn spontaneously than those kept at 19°C. Thirdly, animal selection is also a key step for obtaining high efficiency of induced spawning. The current efficiency of induced spawning in all three lancelets is relatively low (around 30%), which indicated most of selected animals might be actually not ready for spawning. On the other hand, we also noticed that a substantial portion (about 45%, see Figure 5a) of animals tend to spawn spontaneously before heat shock even under constant low temperature, hinting lots of animals with mature gonads were missed in our selection for the induction. Collectively, only 8% of females and 5% of males reared to mature were induced to spawn in our experiments (Figure 5a). In order to increase spawning efficiency in future, we need to find out better morphological characteristics for accurately judging the developmental stages of gonads in amphioxus, particularly indicative characters of fully mature gonads.

Supporting Information

Figure S1. Side-by-side induction experiments using two different temperature-shift methods. Spawning percentage is the number of spawned animals (males or females separately) over the total number of temperature shocked animals (males or females separately) in each examined date. Inductions using Method I (shifting from 19°C to 27°C) and II (shifting from 22°C to 27°C) are shown separately. Inductions of the two cohorts of animals are respectively marked by black and red bold lines under the horizontal coordinates. Statistical analyses of the data are carried out using SPASS software (version 16.0) and two-tailed chi-squared test.
(TIF)

Table S1. Recurring spawning in *B. japonicum* reared under three different temperatures. The experiment was set on 24th Nov. 2011 and data collection was started from 6th Feb. 2012 when most animals began to develop their gonads. On each data collecting date, animal number in each reproductive phase was recorded. Abbreviations: Dev, Development; Spa, Spawning.
(DOC)

Table S2. Side-by-side spawning induction using two methods on two cohorts of *B. becheri* animals.
(DOC)

Table S3. Spawning induction of *B. belcheri* using Method I and II. Period from 7th June 2011 to 31st October 2011 includes spawning induction records of animals which are reared under Zhang's conditions and shocked using Method I; period from 1st November 2012 to 11th April 2012 includes spawning induction records of animals which are reared under updated conditions and shocked using Method I; and period from 12th April 2012 to 23nd Oct. 2012 includes spawning

induction records of animals reared under updated conditions and shocked using Method II.
(DOC)

Table S4. Spawning induction records of *B. japonicum* in 2011 and 2012.
(DOC)

Acknowledgements

The authors thank Ms. Li Q, Yang X, and Mr. Jun F for their help in amphioxus husbandry, Prof. Damin Luo for his help in statistical analyses, and Dr. Yong Zhu from East Carolina University for language editing to the manuscript. We are also grateful to the anonymous reviewer for his/her helpful comments and suggestions.

Author Contributions

Conceived and designed the experiments: GL YQW. Performed the experiments: GL ZHS. Analyzed the data: GL YQW. Wrote the manuscript: GL YQW.

References

1. Gee H (2001) Deuterostome phylogeny: The context for the origin and evolution of the vertebrates. In: PE Ahlberg. Major events in early vertebrate evolution. London: Taylor and Francis. pp. 1-14.
2. Turbeville JM, Schulz JR, Raff RA (1994) Deuterostome phylogeny and the sister group of the chordates: evidence from molecules and morphology. Mol Biol Evol 11: 648-655. PubMed: 8078403.
3. Blair JE, Hedges SB (2005) Molecular phylogeny and divergence times of deuterostome animals. Mol Biol Evol 22: 2275-2284. doi:10.1093/molbev/msi225. PubMed: 16049193.
4. Philippe H, Lartillot N, Brinkmann H (2005) Multigene analyses of bilaterian animals corroborate the monophyly of Ecdysozoa, Lophotrochozoa, and Protostomia. Mol Biol Evol 22: 1246-1253. doi:10.1093/molbev/msi111. PubMed: 15703236.
5. Delsuc F, Brinkmann H, Chourrout D, Philippe H (2006) Tunicates and not cephalochordates are the closest living relatives of vertebrates. Nature 439: 965-968. doi:10.1038/nature04336. PubMed: 16495997.
6. Putnam NH, Butts T, Ferrier DE, Furlong RF, Hellsten U et al. (2008) The amphioxus genome and the evolution of the chordate karyotype. Nature 453: 1064-1071. doi:10.1038/nature06967. PubMed: 18563158.
7. Paps J, Holland PW, Shimeld SM (2012) A genome-wide view of transcription factor gene diversity in chordate evolution: less gene loss in amphioxus? Brief Funct Genomics 11: 177-186. doi:10.1093/bfgp/els012. PubMed: 22441554.
8. Beaster-Jones L (2012) *Cis*-regulation and conserved non-coding elements in amphioxus. Brief Funct Genomics 11: 118-130. doi:10.1093/bfgp/els006. PubMed: 22402505.
9. Candiani S (2012) Focus on miRNAs evolution: a perspective from amphioxus. Brief Funct Genomics 11: 107-117. doi:10.1093/bfgp/els004. PubMed: 22373649.
10. Dehal P, Satou Y, Campbell RK, Chapman J, Degnan B et al. (2002) The draft genome of *Ciona intestinalis*: insights into chordate and vertebrate origins. Science 298: 2157-2167. doi:10.1126/science.1080049. PubMed: 12481130.
11. Shimeld SM, Holland PW (2000) Vertebrate innovations. Proc Natl Acad Sci U S A 97: 4449-4452. doi:10.1073/pnas.97.9.4449. PubMed: 10781042.
12. Garcia-Fernández J, Holland PW (1994) Archetypal organization of the amphioxus *Hox* gene cluster. Nature 370: 563-566. doi:10.1038/370563a0. PubMed: 7914353.
13. Holland LZ, Laudet V, Schubert M (2004) The chordate amphioxus: an emerging model organism for developmental biology. Cell Mol Life Sci 61: 2290-2308. PubMed: 15378201.
14. Koop D, Holland LZ (2008) The basal chordate amphioxus as a simple model for elucidating developmental mechanisms in vertebrates. Birth Defects Res C Embryo TODAY 84: 175-187. doi:10.1002/bdrc.20128. PubMed: 18773463.
15. Bertrand S, Escriva H (2011) Evolutionary crossroads in developmental biology: amphioxus. Development 138: 4819-4830. doi:10.1242/dev.066720. PubMed: 22028023.
16. Yu JK, Holland LZ (2009) Cephalochordates (amphioxus or lancelets): a model for understanding the evolution of chordate characters. Cold Spring Harb Protoc 2009: pdb emo130.
17. Wang YQ, Zhang QJ, Lv XM, Zhong J, Sun Y (2006) Laboratory culturing and acquirement of the second filial generation of amphioxus. Zool Sci 27: 631-634.
18. Zhang QJ, Sun Y, Zhong J, Li G, Lü XM et al. (2007) Continuous culture of two lancelets and production of the second filial generations in the laboratory. J Exp Zool B Mol Dev Evol 308: 464-472. PubMed: 17497691.
19. Xu QS, Ma F, Wang YQ (2005) Morphological and 12S rRNA gene comparison of two Branchiostoma species in Xiamen waters. J Exp Zool B Mol Dev Evol 304: 259-267. PubMed: 15791653.
20. Zhang QJ, Zhong J, Fang SH, Wang YQ (2006) *Branchiostoma japonicum* and *B. belcheri* are distinct lancelets (Cephalochordata) in Xiamen waters in China. Zool Sci 23: 573-579. doi:10.2108/zsj.23.573. PubMed: 16849846.
21. Wang YQ, Fang SH (2005) Taxonomic and molecular phylogenetic studies of amphioxus: a review and prospective evaluation. Zool Res 26: 666-672.
22. Stokes D, Holland ND (1991) Reproduction of the Florida lancelet (*Branchiostoma floridae*): spawning patterns and fluctuations in gonad indexes and nutritional reserves. Invertebra Biol 115: 349-359.
23. Fuentes M, Benito E, Bertrand S, Paris M, Mignardot A et al. (2007) Insights into spawning behavior and development of the European amphioxus (*Branchiostoma lanceolatum*). J Exp Zool B Mol Dev Evol 308: 484-493. PubMed: 17520703.
24. Fuentes M, Schubert M, Dalfo D, Candiani S, Benito E et al. (2004) Preliminary observations on the spawning conditions of the European amphioxus (*Branchiostoma lanceolatum*) in captivity. J Exp Zool B Mol Dev Evol 302: 384-391. PubMed: 15287102.
25. Theodosiou M, Colin A, Schulz J, Laudet V, Peyrieras N et al. (2011) Amphioxus spawning behavior in an artificial seawater facility. J Exp Zool B Mol Dev Evol 316: 263-275. PubMed: 21271675.
26. Li G, Yang X, Shu Z, Chen X, Wang Y (2012) Consecutive spawnings of Chinese amphioxus, *Branchiostoma belcheri*, in captivity. PLOS ONE 7: e50838. doi:10.1371/journal.pone.0050838. PubMed: 23251392.
27. Xu QS, Ma F, Wang YQ (2005) Morphological and *12S rRNA* gene comparison of two *Branchiostoma* species in Xiamen waters. J Exp Zool B Mol Dev Evol 304: 259-267. PubMed: 15791653.
28. Li W, Zhong J, Wang Y (2013) Genetic diversity and population structure of two lancelets along the coast of china. Zool Sci 30: 83-91. doi:10.2108/zsj.30.83. PubMed: 23387841.
29. Wu SC (1986) The early development of amphioxus. In: Z Ganguan. Advances in Science of China Biology. New York: John Wiley. pp. 231-266.
30. Holland ND, Holland LZ (1989) Fine structural study of the cortical reaction and formation of the egg coats in a lancelet (=amphioxus), *Branchiostoma floridae* (phylum Chordata: subphylum Cephalochordata = Acrania). Biol Bull 176: 111-122. doi:10.2307/1541578.
